# Contemporary Strategies of Gene and Cell Therapy in the Treatment of Peripheral Nervous System Injuries and Disorders

**DOI:** 10.3390/ijms27052335

**Published:** 2026-03-02

**Authors:** Alexandra Sharshakova, Valeriya Solovyeva, Galina Masgutova, Alisa Fattakhova, Albert Rizvanov, Albert Sufianov, Galina Sufianova, Ruslan Masgutov

**Affiliations:** 1 Institute for Fundamental Medicine and Biology, Kazan Federal University, 420008 Kazan, Russia; alsharshakova@kpfu.ru (A.S.); vavsoloveva@kpfu.ru (V.S.); gamasgutova@kpfu.ru (G.M.); alisashajmardanova@kpfu.ru (A.F.); 2 Division of Medical and Biological Sciences, Tatarstan Academy of Sciences, 420111 Kazan, Russia; 3 Department of Neurosurgery, Sechenov First Moscow State Medical University of the Ministry of Health of the Russian Federation (Sechenov University), 119991 Moscow, Russia; sufianov_a_a@staff.sechenov.ru; 4 The Research and Educational Institute of Neurosurgery, Peoples’ Friendship University of Russia (RUDN), 117198 Moscow, Russia; 5 Department of Pharmacology, Tyumen State Medical University, 625023 Tyumen, Russia; sufarm@mail.ru; 6 Pestrechinsky Central District Hospital, 422770 Pestrecy, Russia; masgut@gmail.com

**Keywords:** peripheral nervous system, nerve injury, neuropathy, regeneration, neurotrophic factors, gene therapy, cell therapy, Schwann cells, mesenchymal stromal cells, tissue engineering

## Abstract

Injuries and diseases of the peripheral nervous system (PNS) often result in irreversible functional deficits. Current therapeutic approaches demonstrate limited efficacy, which has driven the development of regenerative medicine strategies. This review systematizes contemporary gene and cell therapy approaches aimed at PNS repair and regeneration. Key neurotrophic factors (NGF, BDNF, GDNF, VEGF, etc.) and the molecular mechanisms underlying their regenerative effects are discussed. Gene delivery strategies employing viral and plasmid vectors are analyzed, along with the therapeutic application of various cell populations, including Schwann cells, mesenchymal stromal cells, and derivatives of induced pluripotent stem cells. Particular attention is given to combined gene–cell-based approaches, which enable localized and sustained expression of therapeutic molecules. The integration of advances in genetic engineering, cell biology, and tissue engineering is shaping a new treatment paradigm focused on pathogenetic restoration of nerve tissue. These promising strategies pave the way toward achieving complete functional regeneration following PNS injuries.

## 1. Introduction

The peripheral nervous system (PNS) comprises all neural structures outside the brain and spinal cord, facilitating communication between the central nervous system (CNS) and the body [1]. It includes the somatic nervous system, responsible for sensory perception and voluntary control of skeletal muscles, and the autonomic nervous system, which regulates internal organ function and maintains homeostasis [2,3,4].

Traumatic injuries of the PNS represent a significant medical and social problem due to the high rate of patient disability and substantial economic costs associated with treatment and rehabilitation [5,6]. Peripheral nerve injuries (PNIs) may result from traumatic events (penetrating injuries, compression, traction) as well as non-traumatic causes, such as chronic compression. According to epidemiological studies, the incidence of PNIs in developed countries reaches 13–23 cases per 100,000 individuals per year [7,8]. Such injuries lead to autonomic, motor, and sensory dysfunctions and are often accompanied by muscle atrophy and chronic pain syndromes. Damage to major nerve trunks has particularly severe consequences, significantly reducing patients’ quality of life. In addition to physical impairments, patients with PNIs frequently experience psychosocial challenges, including depressive disorders, chronic stress, difficulties with employment, and social maladaptation, often exacerbated by disability status assignment [9].

Peripheral neuropathies constitute a broad group of disorders, both acquired and inherited, and represent a major medical and social burden. The most common acquired form is diabetic peripheral neuropathy, which develops in approximately 50% of patients with diabetes mellitus. This condition is associated with chronic pain, autonomic dysfunction, and a marked reduction in quality of life, placing a substantial burden on healthcare systems and leading to loss of work capacity [10,11].

Among the inherited forms, Charcot–Marie–Tooth disease (CMT) predominates, with a prevalence of approximately 1:2500. The disease is characterized by distal muscle weakness and atrophy, as well as sensory disturbances, which negatively affect patients’ quality of life and psycho-emotional well-being [12]. In addition to CMT disease, inherited PNS disorders include hereditary neuropathy with liability to pressure palsies, hereditary sensory and autonomic neuropathies, distal hereditary motor neuropathies, and rare syndromic forms such as giant axonal neuropathy [13]. CMT itself is genetically highly heterogeneous and is associated with mutations in more than 100 genes, with the most common being *PMP22*, *MPZ*, *GJB1*, and *MFN2* [14]. Other inherited PNS disorders are caused by mutations in genes such as *SPTLC1*, *NTRK1*, *SCN9A*, *HSPB1*, *HSPB8*, *GARS1*, and *GAN*, reflecting the broad molecular diversity underlying hereditary neuropathies [13]. Currently, therapy for peripheral neuropathies is largely limited to symptomatic treatment, which stimulates active research into novel therapeutic approaches, particularly gene therapy strategies [11].

In the context of traumatic PNIs, the high frequency of unsatisfactory functional outcomes necessitates prolonged and complex rehabilitation programs. Although various surgical approaches have been developed to restore motor and sensory functions and alleviate neuropathic pain, conventional microsurgical techniques remain technically demanding, require a high level of surgical expertise, and are associated with several limitations, including incomplete functional recovery and donor site morbidity. These challenges have driven extensive research and clinical trials aimed at improving existing surgical strategies and, importantly, have stimulated the development of novel therapeutic approaches, including advanced microsurgical techniques and alternative approaches such as gene therapy and cell-based therapies utilizing mesenchymal stromal/stem cells (MSCs) and Schwann cells (SCs) [8,9].

This review will examine the latest gene therapy and cell therapy approaches for PNS injuries and diseases, emphasizing their differing aims, delivery requirements, and challenges when targeting regeneration versus pathology. We first discuss the existing therapeutic approaches and their limitations, then examine emerging gene and cell therapy strategies, followed by combined approaches, and finally summarize the current challenges and future directions.

## 2. Current Treatment Approaches and Their Limitations

### 2.1. Surgical Approaches

**Figure 1:**
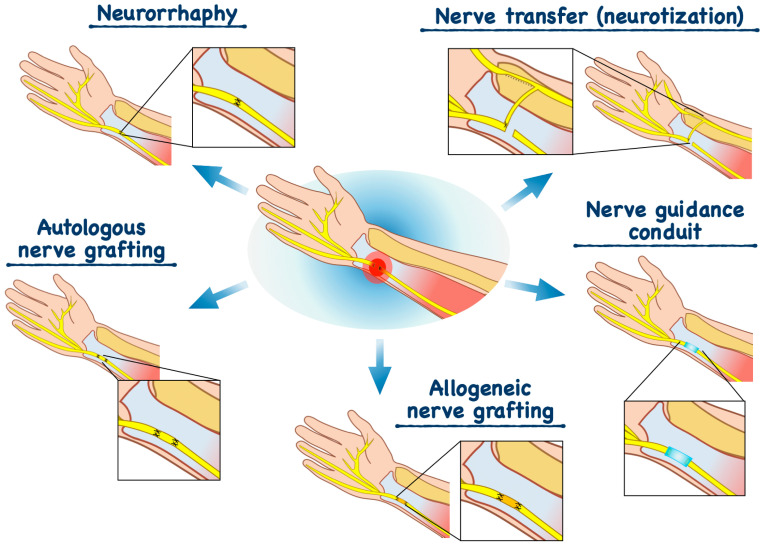
Surgical approaches for the treatment of peripheral nervous system injuries.

To date, surgical intervention remains the primary treatment modality for PNIs. These approaches include direct nerve repair (neurorrhaphy), nerve grafting using autografts or allografts, and the application of tissue-engineered constructs, such as artificial nerve guidance conduits (Table 1 and Figure 1) [15].

Neurorrhaphy is the method of choice for short nerve gaps (<1 cm) and involves direct suturing of the proximal and distal nerve stumps following the resection of nonviable tissue and precise alignment of nerve fascicles and blood vessels [8,17]. Although this approach provides conditions favorable for regeneration, its effectiveness in proximal nerve injuries is limited. This limitation arises because regenerating axons must reach target muscle fibers within a critical time window; otherwise, irreversible denervation occurs. Consequently, a major drawback of neurorrhaphy is the lack of guaranteed complete functional recovery. Additional limitations include partial neuronal loss, reduced synthesis of neurotrophic factors, and delayed axonal regeneration. Precise matching of motor and sensory fascicles is a critical determinant of success, as mismatching leads to impaired reinnervation, prolonged muscle denervation, and subsequent muscle atrophy [16,17].

Autologous nerve grafting is employed in cases of extensive nerve defects (>3 cm), as well as in critical or proximal injuries. Autologous donor nerves commonly include the sural nerve, the superficial cutaneous nerve of the forearm, or the lateral femoral cutaneous nerve. Among these, the sural nerve is most widely used in clinical practice due to its accessibility and acceptable functional cost associated with the donor-site deficit [8,18]. Autografting is considered the “gold standard” for nerve defect reconstruction as autografts provide a natural scaffold for axonal growth through the preservation of endoneurial architecture, SCs, adhesion molecules, and neurotrophic factors while avoiding immune rejection [8,16,19]. However, this approach has several significant limitations, including the limited availability of donor material, the need for additional surgical exposure, the risk of chronic pain, neuroma formation, and permanent sensory loss at the donor site, as well as potential mismatches in graft diameter and fascicular architecture. In extensive injuries, the length and thickness of available autografts are often insufficient to achieve complete functional recovery [8,16,20].

Allogeneic nerve grafting is considered an alternative to autografting, as allografts can be harvested and preserved in tissue banks, eliminating the need for donor nerve harvesting from the recipient and providing an appropriate structural scaffold for regeneration. This approach is typically applied in critical nerve defects (generally ≥3 cm) where the required graft length exceeds the capacity of autologous donation. In such cases, donor or cadaveric nerves are used [8,16,21,22]. The advantages of allografts include preservation of the endoneurial structure, which supports guided axonal regeneration, as well as the presence of donor SCs that facilitate remyelination. However, the major limitation of allogeneic grafts is their high antigenicity, necessitating prolonged immunosuppressive therapy (up to 24 months) to prevent graft rejection. Accordingly, the need for systemic immunosuppression, associated risks (including opportunistic infections and malignancies), and high treatment costs significantly limit the clinical application of this approach. These drawbacks have driven the development of artificial nerve guidance conduits [21].

Nerve transfer (neurotization) is used in severe, particularly proximal injuries associated with the loss of both sensory and motor function, where direct repair or autografting is not feasible [16,23]. This technique involves connecting the proximal end of an intact, functionally less critical donor nerve to the distal stump of the injured nerve. This strategy enables distal-level reinnervation, shortens the axonal regeneration distance, and accelerates functional recovery [16,23]. Nerve transfer is widely employed in brachial plexus injuries, upper limb nerve injuries, and facial nerve reinnervation procedures [23]. The advantages of this method include faster recovery due to the proximity of the donor nerve to target muscles, the possibility of performing surgery outside scarred tissue zones, and preservation of nerve anatomy and biomechanics. However, the approach requires a high level of surgical expertise, is associated with high costs, offers a limited selection of suitable donor nerves, and carries the risk of functional loss in the donor nerve territory [16,23].

Nerve guidance conduits are tubular structures that serve as a “bridge” between the proximal and distal nerve stumps, directing axonal regeneration [8,16,23]. Their mechanism of action is based on the accumulation of axoplasm and tissue fluid from the nerve ends, which promotes the formation of a fibrin matrix that supports cell migration [23]. Conduits may be synthetic (biodegradable or non-resorbable) or biological in origin. To enhance regenerative outcomes, the inner surfaces of conduits can be functionalized with neurotrophic factors, proteins, or extracellular matrix components [8,16,37]. The main advantages of nerve conduits include the elimination of donor-site morbidity, the creation of a controlled microenvironment, the formation of a barrier against infiltration by surrounding tissues, and the establishment of favorable conditions for cell adhesion, migration, and guided axonal growth [16,24]. Despite satisfactory outcomes in the treatment of small nerve gaps, the effectiveness of nerve conduits in extensive nerve defects remains limited. Moreover, hollow conduits may permit disorganized axonal growth, reducing the likelihood of accurate target reinnervation and increasing the risk of aberrant innervation [24].

Despite being the standard of care, surgical approaches for PNIs are limited by incomplete functional recovery, donor-site morbidity, immunological complications, restricted graft availability, and suboptimal outcomes in large or proximal defects.

### 2.2. Physiotherapy

Physiotherapeutic interventions play an important role in rehabilitation programs aimed at improving outcomes following PNIs. These approaches include electrical stimulation (ES), ultrasound therapy, photobiomodulation, and various forms of aerobic exercises (Table 1) [26].

ES is widely used to address neuromuscular impairments, as low-frequency stimulation promotes nerve fiber regeneration. In addition, this modality is effective in preventing and treating muscle atrophy resulting from denervation associated with PNIs [8,16]. The regenerative effects of ES are attributed to the generation of an endogenous electric field at the injury site, under which regenerative factors, such as nerve growth factor (NGF), migrate toward the cathode. This process creates a concentration gradient that guides axonal growth cones and stimulates axonal regeneration [26]. Despite its proven efficacy, ES has several limitations. Its widespread clinical use is constrained by increased operative time, high equipment costs, and incompatibility with regional and local anesthesia, as sodium channel blockers used in these settings may negate the beneficial effects of stimulation. Furthermore, ES has been shown to reduce muscle excitability and muscle fiber cross-sectional area, as well as impair neuromuscular junction integrity and decrease the expression of neural cell adhesion molecule (NCAM) [27].

Ultrasound therapy affects cells through three primary mechanisms: mechanical (alteration of ion channel activity and nerve impulse transmission), thermal (conversion of acoustic energy into heat), and cavitational (formation and oscillation of gas bubbles that interact with tissues). Bubble oscillations induce cellular deformation, leading to changes in biochemical signaling pathways and, consequently, alterations in cell morphology and function [26]. Numerous preclinical and clinical studies indicate that low-intensity ultrasound promotes peripheral nerve regeneration by increasing axon number and diameter, enhancing myelination, accelerating nerve conduction velocity, and modulating the expression of neurotrophic factors. Nevertheless, the widespread implementation of this approach is limited by an incomplete understanding of its molecular and cellular mechanisms, as well as the need for further studies to establish clinical efficacy and standardize therapeutic protocols [28].

Photobiomodulation (low-level laser therapy) is frequently used as an adjunct modality in postoperative rehabilitation. This technique is based on the absorption of light by mitochondrial enzymes, primarily cytochrome c oxidase, which activates the respiratory chain and enhances adenosine triphosphate (ATP) synthesis. Increased cellular energy availability, in turn, stimulates DNA and RNA synthesis [26,29]. This effect is particularly relevant in PNIs, where elevated energy demands under conditions of metabolic stress may lead to cell death and neurodegeneration. Photobiomodulation supports peripheral nerve repair by enhancing mitochondrial function, stimulating the production of growth factors (e.g., NGF), modulating immune responses, and promoting angiogenesis. However, study outcomes remain inconsistent, largely due to variability in irradiation parameters (wavelength, intensity, exposure duration), and optimal treatment protocols have yet to be established [26,30].

PNIs not only cause local impairments but may also induce the apoptosis of spinal motor neurons. This disrupts connectivity between upper and lower motor neurons, ultimately impairing the transmission of motor commands to muscles and sensory information to the brain [26]. Aerobic exercise has been shown to enhance blood flow, improve tissue oxygenation, stimulate the release of neurotrophic factors, and activate specific neuronal pathways, thereby contributing to functional recovery of the nervous system [26,32]. Experimental evidence suggests that intrinsic regenerative capacity is higher in young animals, and physical activity further accelerates this process, whereas in adult organisms, the regenerative benefits of exercise are less pronounced [31]. The positive effects of aerobic exercise on peripheral nerve regeneration have been convincingly demonstrated in preclinical studies; however, clinical evidence remains limited. Existing reports indicating beneficial effects of physical activity in diabetic neuropathy require confirmation in future controlled clinical trials [33].

Thus, the principal limitation of physiotherapeutic approaches in the treatment of PNIs is the lack of standardized and universally accepted protocols. These interventions cannot be considered as standalone therapies, particularly in cases of extensive nerve damage; however, they have demonstrated significant supportive benefits when applied in combination with surgical treatment strategies, contributing to improved functional recovery [38,39].

### 2.3. Pharmacotherapy

At present, there are no approved pharmacological agents that directly target the etiopathogenesis of PNIs, inherited neuropathies, or diabetic neuropathy (Table 1). Current pharmacotherapy is largely limited to symptomatic management; nevertheless, several disease-modifying agents are under development and undergoing preclinical and clinical evaluation [40].

Erythropoietin has emerged as a promising candidate for enhancing nerve tissue regeneration and providing neuroprotection. It has been demonstrated that erythropoietin exerts pronounced neuroprotective effects in PNIs by preventing axonal degeneration and neuronal apoptosis. A major challenge associated with erythropoietin therapy is dose optimization, as its potent neuroprotective activity is accompanied by strong hematopoietic effects, which may lead to adverse outcomes. Consequently, the development of erythropoietin derivatives lacking hematopoietic activity, such as carbamylated erythropoietin, has attracted considerable interest, as these compounds retain neuroprotective potential. Despite encouraging preclinical data, further studies are required before erythropoietin and its analogues can be translated into clinical practice for PNI treatment, including the determination of optimal dosing regimens, therapeutic windows, and a deeper understanding of their molecular mechanisms of action [34].

Steroid hormones, particularly estrogen and progesterone, also exhibit neuroprotective properties by promoting remyelination and peripheral nerve regeneration [29]. Similarly, corticosteroids (including hydrocortisone, dexamethasone, and prednisolone) have demonstrated beneficial effects on the recovery of sensory and motor functions following PNIs and are also used in the management of chemotherapy-induced, metabolic, and compression neuropathies. However, the broad pharmacological activity of steroids is associated with a substantial risk of serious adverse effects, particularly with long-term administration and high-dose regimens. These include osteoporosis, hyperglycemia, arterial hypertension, gastrointestinal mucosal ulceration, impaired wound healing, and suppression of the hypothalamic–pituitary–adrenal axis [8,35].

4-Aminopyridine is a potassium channel blocker approved by the U.S. Food and Drug Administration (FDA) for the symptomatic treatment of multiple sclerosis [41]. Its mechanism of action involves the enhancement of neuromuscular transmission through prolongation of the action potential, increased calcium influx into presynaptic terminals, and, consequently, augmented neurotransmitter release. In preclinical models of PNIs, 4-aminopyridine has been shown to promote remyelination, improve nerve conduction velocity, and accelerate functional recovery. However, its clinical application is limited by a narrow therapeutic window and the risk of adverse effects, including tremor, anxiety, and seizure activity [8,29,36].

The broad clinical application of pharmacological therapies for PNIs is limited by dose-dependent adverse effects, which necessitate the development of standardized and optimized dosing protocols. Nevertheless, pharmacotherapy has demonstrated considerable efficacy as an adjunct to surgical intervention, as well as in combinatorial regimens involving multiple pharmacological agents, leading to enhanced functional recovery [42,43].

#### 2.3.1. Regenerative Potential of Trophic Growth Factors

The use of recombinant growth factors in the treatment of peripheral neuropathy is associated with several limitations, despite their significant therapeutic potential. Growth factors such as vascular endothelial growth factor (VEGF), brain-derived neurotrophic factor (BDNF), NGF, and others have demonstrated the ability to stimulate reparative nerve regeneration by improving microcirculation, trophic support, and myelination. However, their rapid degradation in the systemic circulation and target tissues markedly reduces therapeutic efficacy and necessitates repeated administration, thereby increasing the risk of adverse effects (Table 2) [44].

##### Vascular Endothelial Growth Factor

**Figure 2:**
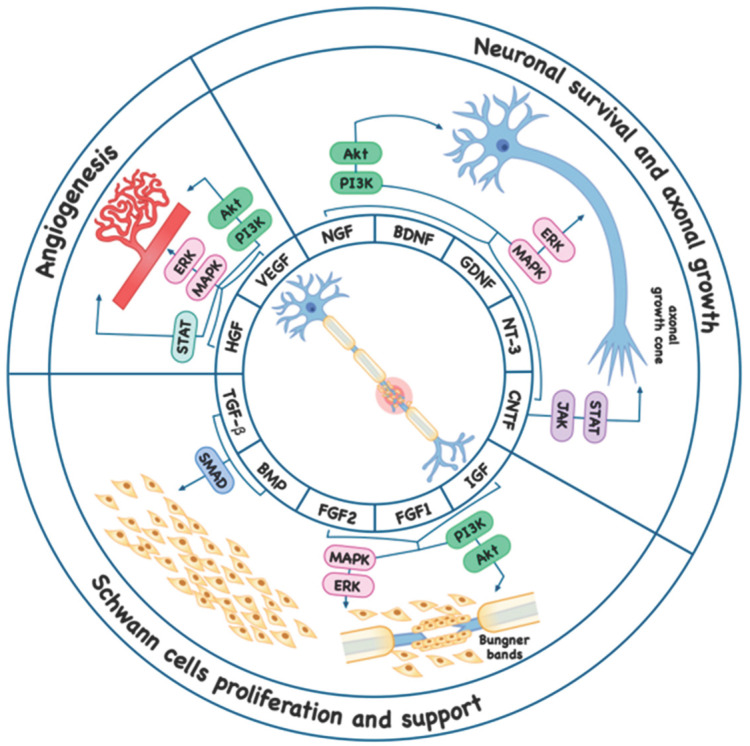
Mechanisms of action of growth factors in peripheral nerve regeneration and their signaling pathways. Abbreviations: PI3K/Akt: Phosphatidylinositol-3-kinase/Protein kinase B, MAPK/ERK: Mitogen-activated protein kinase/Extracellular signal-regulated kinase, JAK/STAT: Janus-associated kinase/Signal transducer and activator of transcription, SMAD: Suppressor of mother against decapentaplegic.

VEGF is a key mediator that plays a multimodal role in recovery processes following PNI. Accumulating evidence indicates that VEGF is directly involved not only in the stimulation of angiogenesis but also exerts direct neurotrophic and neuroprotective effects that promote axonal regeneration (Figure 2) [70].

The biological effects of VEGF during nerve regeneration are mediated through several interconnected mechanisms. In addition to enhancing neovascularization, which improves trophic support at the site of injury, VEGF directly stimulates SC proliferation and migration [60]. An important aspect of its activity is the modulation of the local molecular environment: VEGF enhances the expression of neurotrophic factors such as NGF and BDNF. Simultaneously, VEGF participates in the regulation of the immune response by increasing vascular permeability and facilitating macrophage recruitment to the injury site, thereby accelerating debris clearance and creating a microenvironment favorable for regeneration [69].

Of particular interest is the synergistic interaction between VEGF and other growth factors, especially fibroblast growth factor-2 (FGF2). It has been shown that FGF2 (basic fibroblast growth factor) enhances VEGF synthesis, and their combined application results in a significantly stronger regenerative response compared with the effects of either factor alone, indicating the involvement of complementary signaling pathways [60].

Preclinical studies in models of peripheral neuropathy confirm the multifaceted reparative potential of VEGF. Its administration promotes revascularization, remyelination, and structural axonal restoration, which collectively lead to improved functional outcomes (Table 2) [86].

##### Fibroblast Growth Factor

FGF2 is a polypeptide that regulates key cellular processes, including proliferation, survival, migration, and differentiation. It plays an important role in stimulating angiogenesis, fibroblast proliferation, and neuronal repair [60]. FGF1 (acidic fibroblast growth factor) is involved in similar processes, including angiogenesis, neurogenesis, cellular differentiation, and the regulation of apoptosis (Figure 2) [62].

Both factors exhibit increased expression in injured nerves following axotomy. Local application of FGF2 promotes sensory neuron regeneration and axonal growth [87], whereas its deficiency disrupts axonal maturation and remyelination processes [60]. FGF1, studied in animal models of peripheral neuropathy and complete spinal cord transection in combination with nerve grafts, demonstrates pronounced neuroprotective and regenerative effects by enhancing axonal growth and contributing to the recovery of motor function (Table 2) [47,88].

In preclinical studies, FGF2 has shown high efficacy when delivered using biomaterial-based carriers as well as in combination with NGF. These strategies promoted the regeneration of myelinated fibers, SC proliferation, remyelination, and restoration of motor function [48,89,90,91].

##### Nerve Growth Factor

NGF plays a fundamental role in maintaining the viability, differentiation, and regenerative capacity of sensory and sympathetic neurons (Table 2). NGF exerts its pro-regenerative effects through interaction with its receptors tropomyosin receptor kinase A (TrkA) and p75 neurotrophin receptor (p75NTR), promoting neuronal survival, axonal outgrowth, myelin debris clearance, and modulation of Schwann cell migration, thereby facilitating extracellular matrix remodeling and creating a microenvironment conducive to nerve regeneration [46,47,48].

The clinical translation of systemically administered recombinant human NGF is associated with significant challenges. A large phase III clinical trial in diabetic neuropathy demonstrated limited efficacy and dose-limiting adverse effects, including hyperalgesia [92]. In contrast, success has been achieved with local delivery approaches: in 2018, the FDA approved recombinant NGF-based eye drops (Oxervate™) for the treatment of neurotrophic keratopathy [93,94].

A promising direction involves the development of controlled NGF delivery systems in combination with bioengineered constructs. In a rat sciatic nerve injury model, the use of chitosan and poly(lactide-co-glycolide) (PLGA) microspheres loaded with NGF significantly enhanced nerve regeneration and prevented muscle atrophy [95]. Additional strategies have also demonstrated efficacy, including biocompatible conductive nerve conduits with ultrasound-triggered NGF release, which promoted the recovery of motor function [96], as well as nerve conduits filled with hydrogels based on bioconjugated hyaluronic acid and chitosan. These systems provided sustained NGF delivery, and in the repair of a 10-mm nerve defect, resulted in improved remyelination and functional recovery [97].

##### Brain-Derived Neurotrophic Factor

BDNF is a key regulator of nervous system plasticity and homeostasis. By binding to the TrkB receptor, BDNF promotes the differentiation and survival of neurons and glial cells in both the CNS and PNS, and regulates myelination, neuronal migration, and axon pruning (Table 2 and Figure 2) [48].

Preclinical studies in models of peripheral neuropathy demonstrate a substantial regenerative potential of BDNF. A meta-analysis comprising 40 animal studies showed that BDNF-based therapies significantly improved axonal regeneration, myelination, and functional recovery compared with control groups. The highest efficacy was observed with combinatorial strategies, such as the co-administration of BDNF with stem cells or other neurotrophic factors [51].

However, the clinical translation of BDNF faces considerable limitations. Clinical trials in amyotrophic lateral sclerosis (ALS) failed to demonstrate sustained therapeutic benefits. One of the major challenges is the unfavorable pharmacokinetic profile and low tissue penetration of recombinant BDNF, which hinder its efficient delivery to target cells [54].

##### Glial Cell Line-Derived Neurotrophic Factor

Glial cell line-derived neurotrophic factor (GDNF) is a multipotent cytokine that plays a critical role in the survival, development, and plasticity of diverse neuronal populations in both the CNS and PNS. Its principal cellular targets include spinal motor neurons and midbrain dopaminergic neurons (Table 2). The biological effects of GDNF are mediated through its binding to the GFRα1 co-receptor and activation of the RET receptor complex, leading to enhanced neuronal survival, axonal growth, and support of regenerative processes following nerve injury (Figure 2) [48,55].

The biological significance of GDNF extends beyond neuronal survival and includes active involvement in regeneration and neuroplasticity. Experimental studies have demonstrated that GDNF enhances axonal myelination following injury, stimulates neurite outgrowth and branching, and modulates synaptic organization, including the remodeling of neuromuscular junctions [48,56]. In the context of peripheral neuropathies, particularly severe forms of PNI, GDNF-based therapy in animal models has shown a comprehensive neuroprotective and neuroregenerative effect. Observed outcomes include increased motor neuron survival, regeneration of motor axons with subsequent reinnervation of target muscles, and significant improvement in motor function [47,56,98].

Despite compelling preclinical evidence, the translation of GDNF into clinical practice has faced significant limitations. In randomized controlled trials involving patients with Parkinson’s disease, intracerebral administration of GDNF failed to produce statistically significant clinical improvements in primary outcome measures [99]. These findings highlight the need for further investigation of GDNF pharmacokinetics, optimization of delivery strategies, and the development of more selective therapeutic approaches.

##### Ciliary Neurotrophic Factor

Ciliary neurotrophic factor (CNTF) is classified as a neuropoietic cytokine and plays a key role in maintaining nervous system homeostasis. Its biological activity is associated with supporting the survival of various neuronal populations and modulating the functional state of glial cells (Table 2 and Figure 2) [57,58].

In the PNS, CNTF is predominantly expressed and accumulated in the cytoplasm of SCs, where it functions as an injury-responsive factor. Levels of CNTF protein and its mRNA are markedly upregulated in response to nerve injury, with expression dynamics closely correlating with phases of demyelination and subsequent remyelination. The release of CNTF from injured SCs is considered an important early neuroprotective mechanism that establishes a supportive microenvironment for regenerating axons [57].

Experimental studies in models of PNIs confirm the therapeutic potential of CNTF. The application of bioactive composite nerve conduits incorporating CNTF significantly enhanced axonal regeneration, stimulated SC proliferation, and led to improved functional recovery [100]. Moreover, combination therapy strategies in which CNTF is administered together with other growth factors demonstrate synergistic effects, resulting in accelerated nerve regeneration and more pronounced functional recovery [48,101].

Despite encouraging preclinical results, particularly with respect to motor neuron support, the clinical translation of CNTF has encountered notable challenges. In controlled clinical trials for ALS, CNTF therapy proved to be safe but failed to show a statistically significant clinical benefit in patients [49,102]. These outcomes underscore the complexity of neuroprotective interventions in progressive neurodegenerative diseases and highlight the need to optimize delivery methods, dosing regimens, and potentially implement multifactorial therapeutic approaches.

##### Insulin-Like Growth Factor-1

Insulin-like growth factor-1 (IGF-1) is a neurotrophic factor that plays a crucial role in maintaining the viability and plasticity of motor, sensory, and sympathetic neurons. Its physiological functions include the promotion of neuronal survival, enhancement of axonal growth cone motility, and suppression of apoptotic processes (Table 2) [48]. In the context of the PNS, IGF-1 exerts pleiotropic effects on SCs, acting as a potent mitogen. It induces their proliferation, migration, and differentiation and plays a critical role in myelination while simultaneously inhibiting SC apoptosis [65,103].

IGF-1 exerts its effects through activation of the IGF-1 receptor, regulating Schwann cell proliferation and differentiation and thereby supporting myelination and functional nerve regeneration following injury (Figure 2) [65,103].

The therapeutic potential of IGF-1 in PNI repair has been confirmed in experimental models. Targeted delivery strategies, such as the encapsulation of IGF-1 into nanoparticles or its incorporation into hydrogel systems in combination with VEGF, have demonstrated significant regenerative effects. These approaches promoted accelerated reinnervation of target muscles and resulted in marked improvement of functional recovery in animal models with PNIs [68,104].

##### Neurotrophin-3

Neurotrophin-3 (NT-3), a member of the neurotrophin family, is a low-molecular-weight secreted protein that plays a key role in the development and maintenance of the PNS. Its biological effects include the regulation of neuronal morphology, support of SC survival and functional activity, and control of directed axonal growth (Table 2) [48,71]. The primary mechanism of NT-3 action is its binding to the TrkC receptor, which leads to activation of intracellular signaling pathways that are critical for the stimulation of nerve regeneration following injury (Figure 2) [72].

Experimental studies in models of PNIs demonstrate the therapeutic potential of NT-3. Local and controlled delivery of NT-3 using a biodegradable nerve conduit based on a silk–chitosan composite material resulted in significant improvement in functional recovery, supporting the efficacy of combined approaches in creating a favorable microenvironment for regeneration [105]. These findings highlight the promise of NT-3 as a component of tissue-engineered constructs for targeted neuroregenerative applications.

##### Hepatocyte Growth Factor

Hepatocyte growth factor (HGF) is a multifunctional cytokine exhibiting both paracrine and autocrine activity. It is predominantly synthesized by cells of mesenchymal origin. HGF mediates anti-inflammatory and antifibrotic actions, creating conditions conducive to effective tissue repair and regeneration (Figure 2) [74].

Within the PNS, HGF functions as an important neurotrophic agent. It has been shown to enhance neuronal survival and stimulate neurite outgrowth in motor, sensory, and sympathetic neurons (Table 2). The physiological relevance of HGF/c-Met signaling for the development and maintenance of the PNS is further supported by evidence that disruption of this pathway leads to pronounced pathological changes, such as reduced sensory innervation density and structural abnormalities in peripheral nerves [75,76,77]. These findings underscore the critical role of endogenous HGF in maintaining neuronal homeostasis and PNS morphogenesis.

##### Transforming Growth Factor-β

Transforming growth factor-β (TGF-β) is a pleiotropic cytokine that plays a central role in the regulation of cell proliferation and differentiation, wound healing processes, and modulation of the immune response. In the context of PNI, TGF-β acts as a key organizer of the regenerative microenvironment by exerting targeted effects on SC function (Table 2) [78]. In peripheral neuropathy or nerve trauma, TGF-β regulates SC proliferation and promotes the reprogramming of resident SCs toward a reparative, proliferation-activated phenotype (Figure 2). In addition, this cytokine participates in the recruitment of macrophages to the injury site, where phagocytic cells mediate the clearance of myelin debris—an essential prerequisite for accelerated axonal regeneration [79,81].

The therapeutic potential of TGF-β in peripheral nerve repair has been confirmed in experimental studies using tissue-engineered constructs. In particular, Nie et al. developed a composite artificial nerve graft consisting of a chitosan conduit filled with a collagen gel containing SCs and TGF-β1. The efficacy of this construct was evaluated in a rat sciatic nerve transection model with a 10-mm nerve gap. The results demonstrated that application of the composite graft achieved high-quality nerve regeneration comparable to that obtained with autologous nerve grafts and significantly superior to outcomes observed in the empty conduit group. These findings indicate that the local delivery of TGF-β1 effectively activated the transplanted SCs, creating favorable conditions for directed axonal growth and sustained regenerative processes [106].

##### Bone Morphogenetic Protein

Certain members of the bone morphogenetic protein (BMP) family, which structurally belong to the TGF-β superfamily, play critical roles in regulating neuronal differentiation and survival (Figure 2) [82]. In PNIs, increased expression of BMP7 has been observed, and the exogenous administration of BMP7 has demonstrated the ability to support SC survival and proliferation, as well as to stimulate axonal regeneration [81,83]. Another key member of this family, BMP5, plays an important role in neurogenesis and neural tissue repair (Table 2). BMP5 has been shown to reduce neuronal loss in experimental models of Parkinson’s disease, induce the differentiation of neural stem cells into dopaminergic neurons, and stimulate dendritic growth in cultures of sympathetic neurons [84].

Thus, despite the pronounced therapeutic potential of recombinant growth factors demonstrated in preclinical studies, their direct clinical application for the treatment of peripheral neuropathies faces several limitations. These include suboptimal pharmacokinetic properties (short half-life and systemic diffusion) and potential adverse effects associated with systemic exposure. In this context, the development of gene- and cell-based therapeutic strategies represents a promising direction, as such approaches can provide localized, sustained, and physiologically regulated expression of neurotrophic factors at the site of injury, thereby improving the efficacy and safety of regenerative therapies.

## 3. Gene Therapy

Gene therapy for PNS disorders, encompassing both inherited and acquired neuropathies, is a rapidly advancing field and is currently at the preclinical and clinical stages of development. Given that existing therapeutic approaches are largely limited to symptomatic management, gene therapy offers a fundamentally different strategy aimed at addressing the underlying molecular and genetic mechanisms of pathogenesis. This approach opens new prospects for the development of more effective and durable therapeutic solutions [8,107].

A detailed comparison of viral and non-viral vector systems used in gene therapy applications—including cargo capacity, transduction efficiency, immunogenicity, and targeting properties—was comprehensively summarized in a recent review [108]. Among the AAV-based platforms, serotypes AAV9, AAVrh10, and engineered variants such as AAV-PHP.eB and AAV-PHP.S have demonstrated an efficient transduction of PNS tissues, including dorsal root ganglion neurons and SCs [109,110]. For lentiviral systems, vesicular stomatitis Indiana virus glycoprotein G (VSV-G)-pseudotyped human immunodeficiency virus 1 (HIV-1)-based vectors remain widely used for the efficient transduction of primary SCs and dorsal root ganglion neurons in vitro with stable long-term expression [111].

**Figure 3:**
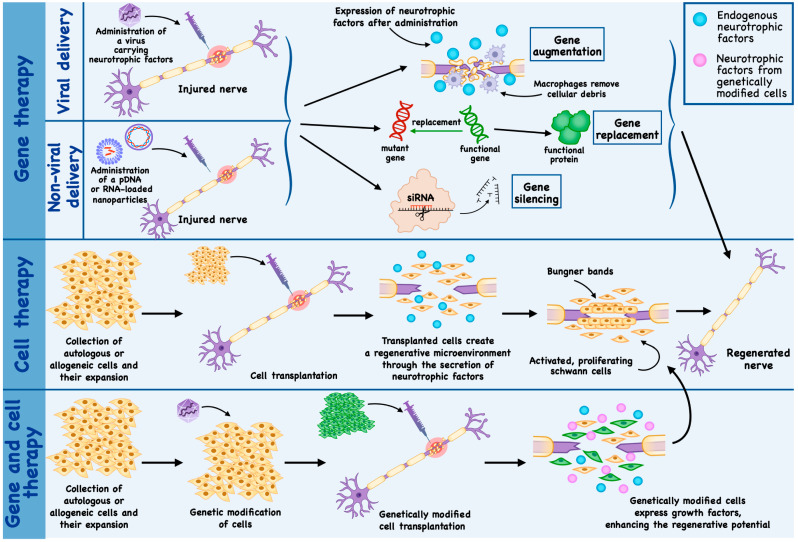
Mechanisms of action of gene, cell-based, and gene–cell therapies in traumatic injuries and diseases of the PNS.

In traumatic injuries of the PNS, the primary objective of gene therapy is to stimulate axonal regeneration and create a permissive environment for axonal growth [8]. In contrast, in inherited peripheral neuropathies, therapeutic strategies are focused on correcting specific genetic defects, with the aim of enhancing SC-mediated myelination, improving mitochondrial function, and restoring impaired axonal transport [107] (Figure 3).

### 3.1. Preclinical Studies of Gene Therapy for Peripheral Nerve Injury

To date, no clinical trials of gene therapy for PNI have been conducted; therefore, current evidence regarding its efficacy is based exclusively on preclinical studies. A promising direction involves the use of gene therapy to regulate and stimulate axonal regeneration through the targeted expression of neurotrophic factors and other growth-promoting molecules. It is well-established that SCs respond to nerve injury by creating a regenerative environment through the secretion of factors such as GDNF, BDNF, and NGF. Genetic delivery of these factors enables the amplification of the intrinsic regenerative capacity of the PNS [8,56,112,113]. However, the therapeutic application of neurotrophic factors is associated with several challenges, including their short half-life, limited tissue penetration, and high biological activity, which may induce adverse effects in non-target tissues [56,71].

The effects of gene therapy using a recombinant replication-deficient adenoviral vector encoding the *β-NGF* gene have been evaluated in a rat model of PNI. Local administration of the vector to the injury site promoted sensory recovery and enhanced nerve regeneration following compressive nerve injury [114].

In preclinical studies, BDNF has been widely investigated as a promising therapeutic agent for PNIs. Several reports have demonstrated the efficacy of combinatorial strategies based on lentiviral delivery of the *BDNF* gene together with genes encoding other neurotrophic factors, resulting in significant improvements in functional recovery in experimental models [51,115,116]. In particular, a recent study addressing the pathogenesis of CMT2D showed that intramuscular administration of adeno-associated virus (AAV) serotype 8 expressing the human *BDNF* gene restored impaired axonal transport, which represents a key pathophysiological defect in this disorder [117]. These findings highlight the therapeutic potential of BDNF-based gene therapy for correcting not only structural damage but also fundamental functional impairments of axons in peripheral neuropathies.

The role of temporally controlled *GDNF* expression has also been investigated using a doxycycline-inducible immune-evasive gene regulation system (dox-i-GDNF). Delivery of the *GDNF* gene via a lentiviral vector to reimplanted ventral spinal nerve roots following avulsion in rats resulted in enhanced motor neuron survival and improved peripheral axon regeneration while avoiding adverse effects associated with uncontrolled gene expression [56]. By comparing regeneration following lumbar versus cervical ventral root avulsion, the authors demonstrated that the efficacy of dox-i-GDNF depends on the length of the regenerative pathway: at shorter distances, controlled GDNF expression promoted successful reinnervation and functional limb recovery, whereas at longer distances, its effectiveness was reduced due to the development of chronic denervation and loss of SC regenerative capacity [56].

Gene therapy using CNTF has been extensively studied as a strategy to stimulate regeneration in the CNS. Major research efforts have focused on optic nerve regeneration and repair following spinal cord injury. Experimental data indicate that gene delivery of *CNTF*, enabling sustained local expression, effectively stimulates the regeneration of injured optic axons, promoting their growth up to the level of the optic chiasm. Moreover, *CNTF* expression has been shown to enhance long-distance regeneration of damaged axons in other regions of the CNS, supporting its potential as a key factor for overcoming the inhibitory environment of the injured central neuronal tract [118,119,120].

A lentiviral vector enabling stable and long-term expression of *FGF2* has also been developed. Evaluation of this construct in animal models of PNI revealed that *FGF2* expression was predominantly localized to the basal membrane of SCs. The use of SCs genetically modified with a lentiviral vector expressing *FGF2*, in combination with a silicone nerve conduit, resulted in a marked enhancement of motor axon regeneration [121]. In another study, the effects of the local injection of a plasmid construct pBud-coVEGF165-coFGF2 into the epineurium of the rat sciatic nerve were investigated. This procedure was confirmed to be safe, and the induced local co-expression of *VEGF165* and *FGF2* promoted the formation of a complex pro-angiogenic and pro-regenerative microenvironment at the injury site, thereby creating favorable conditions for reparative processes [122].

Gene therapy using AAV9-IGF1 has been investigated in mice with a model of spontaneous autoimmune peripheral polyneuropathy. Systemic administration of the *IGF1* gene demonstrated a multifaceted therapeutic effect, including direct neuroprotective activity, objective functional improvement, and modulation of the immune response manifested by the suppression of inflammatory processes within peripheral nerves [123]. In another study conducted in 90 rats with experimental PNIs, local delivery of plasmid DNA (pDNA) encoding *IGF1* using liposomes as a carrier resulted in a significant reduction in motor neuron apoptosis within the corresponding segments of the spinal cord, confirming the pronounced neuroprotective potential of this strategy [124].

In an experimental model of PNI, a combined gene therapy strategy using pDNA encoding *VEGF* and *NGF* was evaluated. This combined approach demonstrated a strong synergistic effect, resulting in statistically significant improvements in axonal growth and functional recovery compared with monotherapy using either factor alone [125].

To achieve targeted delivery of NT-3 in PNIs, advanced nano- and tissue-engineering strategies are being developed. One approach involves the use of exosomes loaded with *NT-3* mRNA, followed by their incorporation into nerve conduits. This strategy was shown to improve the histological morphology of regenerated nerves and accelerate functional recovery [126]. As an alternative method, delivery of *NT-3* mRNA via lipoplexes was employed, enabling prolonged *NT-3* expression in SCs and leading to a statistically significant enhancement of neurite outgrowth in vitro [127].

In a mouse model of traumatic PNI, the effects of intramuscular administration of pDNA encoding the human *HGF* gene were evaluated. Delivery of pDNA via electroporation resulted in accelerated structural and functional nerve recovery. It was demonstrated that HGF indirectly enhances nerve regeneration through the activation of SCs. *HGF* expression in muscle tissue was detectable as early as 72 hours after administration; however, data regarding the duration and stability of this expression remain limited [128].

It was also shown that local administration of an adenoviral vector encoding *BMP7* into the injured sciatic nerve of rats led to improved functional recovery and reduced severity of demyelination and axonal degeneration [82]. In studies examining the effects of lentiviral-mediated *BMP5* delivery on the progression of diabetic neuropathy in mice, BMP5 was found to reduce pain sensitivity and neuronal apoptosis while improving mitochondrial function [84].

### 3.2. Clinical Studies of Gene Therapy for Peripheral Neuropathies and Peripheral Nervous System Disorders

Gene therapy demonstrates significant potential in the treatment of diabetic peripheral neuropathy, a common complication of diabetes mellitus characterized by chronic neuropathic pain. In 2014, a Phase I/II clinical trial of VM202 (Engensis)—a pDNA encoding two isoforms of HGF (NCT01475786)—was completed. Intramuscular administration of VM202 into the calf muscles exhibited a favorable safety and efficacy profile, including a reduction in pain that persisted for up to three months post-injection [129]. These encouraging results provided the rationale for a Phase III trial, in which a regimen of two series of VM202 injections confirmed a favorable safety profile and the absence of serious adverse events (NCT02427464). Although no significant reduction in pain compared to the placebo was observed during the primary 9-month follow-up, an additional 12-month cohort demonstrated clinically meaningful and sustained pain relief (NCT04055090). These conflicting findings underscore the need for another large-scale Phase III study [130].

VM202 has also been investigated in a Phase I/IIa trial for the treatment of CMT1A (NCT05361031). Moreover, its therapeutic potential has been explored in ischemic heart disease and ALS. In these studies, the therapeutic effect of VM202 tended to diminish over time, likely reflecting the transient nature of *HGF* gene expression and, consequently, the temporary action of the therapy [131,132]. According to results from the CMT1A study, intramuscular administration of HGF-encoding pDNA demonstrated safety, good tolerability, and contributed to functional improvement in patients [133].

In another ongoing Phase I/II clinical trial (NCT03520751), gene delivery of *NT-3* is being evaluated in patients with CMT1A associated with duplication of the peripheral myelin protein 22 (PMP22) gene. The study involves intramuscular administration of a self-complementary AAV encoding the complementary DNA (cDNA) of the *NT-3* gene under the control of a muscle-specific promoter (scAAV1.tMCK.NTF3) into the gastrocnemius, tibialis anterior, and rectus femoris muscles of both lower limbs. Participant enrollment has not yet begun, and study results have not been published. Preclinical data in mouse models indicate a high safety profile, absence of adverse effects, and improved functional outcomes, supporting the potential therapeutic efficacy of this approach [134].

In 2013, a Phase I trial of NP2 gene therapy for opioid-resistant pain in cancer patients (NCT00804076) was completed. The therapeutic construct consisted of a herpes simplex virus type 1 (HSV-1)-based vector encoding the preproenkephalin gene, which was administered intradermally. HSV-1 was chosen as the vector due to its natural tropism for peripheral sensory neurons. The gene product, preproenkephalin, inhibits pain transmission by activating δ-opioid receptors expressed on peripheral sensory neurons and secondary neurons in the dorsal horn of the spinal cord. Phase I results demonstrated that NP2 was well-tolerated, safe, and produced pain reduction at medium and high doses. However, due to the small sample size and the absence of a placebo control group, a Phase II trial (NCT01291901) was initiated but subsequently discontinued due to insufficient funding [135,136,137].

Another branch of gene therapy focuses on compensating for defects in genes whose mutations underlie various PNS disorders. Such pathologies include adrenomyeloneuropathy, caused by mutations in the ATP-binding cassette subfamily D member 1 (ABCD1) gene and characterized by the development of peripheral neuropathy [138]. In 2022, a Phase I/II clinical trial of SBT101—a AAV9 vector encoding the human *ABCD1* cDNA—was initiated (NCT05394064). According to preliminary data presented at a conference, intrathecal administration of SBT101 was well-tolerated; however, efficacy assessment remains inconclusive due to disease progression observed in three patients [139]. However, the SBT101 trial was discontinued in late 2025 for business and strategic reasons; no safety concerns were reported (NCT05394064).

In 2015, a Phase I clinical trial was initiated using a self-complementary AAV9 vector expressing the gigaxonin (GAN) gene under the control of a synthetic JeT promoter (scAAV9/JeT-GAN) (NCT02362438). The therapy is administered intrathecally to patients with confirmed giant axonal neuropathy, a rare autosomal recessive neurodegenerative disorder affecting both the CNS and PNS [140]. Observations indicated that gene therapy contributed to improvements in motor function but was associated with several adverse events, as well as elevated anti-AAV9 antibody titers in both serum and cerebrospinal fluid despite the use of immunomodulatory therapy [141,142].

Krabbe disease, caused by mutations in the galactosylceramidase (GALC) gene, leads to demyelination in the CNS and PNS [143,144]. A Phase I/II study evaluated the safety and efficacy of AAVrh.10-based gene therapy encoding *GALC* cDNA (FBX-101, AAVrh.10-hGALC) in children under 12 months of age with Krabbe disease (NCT04693598). The therapy employed a combined approach: following hematopoietic stem cell (HSC) transplantation, FBX-101 was administered systemically via intravenous injection. The vector demonstrated a favorable safety profile and good tolerability, with detectable enzyme activity in both serum and cerebrospinal fluid [145]. Subsequently, the study was expanded to include patients up to 18 years of age (NCT05739643); however, the results have not yet been published.

A noteworthy translational aspect is the registered VEGF-based therapy in the Russian Federation (Neovasculgen), primarily indicated to stimulate angiogenesis to improve regional blood flow and promote wound healing [146]. Considering the critical dependence of peripheral nerves on adequate vascular supply and trophic support, such therapies may exert an indirect beneficial effect on nerve tissue health and reparative processes in clinical practice.

Table 3 provides an overview of clinical trials of gene therapy for PNS disorders.

Collectively, preclinical and early clinical studies demonstrate that gene therapy for PNS disorders can promote axonal regeneration, remyelination, neuroprotection, and functional recovery through the targeted modulation of neurotrophic, angiogenic, metabolic, and immune-related pathways. However, major hurdles remain, including the lack of clinical trials for traumatic PNI, variability and transient nature of transgene expression, delivery efficiency, immune responses to viral vectors, and the need for long-term safety and efficacy data to enable broader clinical translation.

## 4. Cell Therapy

Cell therapy represents one of the most promising areas of regenerative medicine. In the context of PNIs, this approach offers the potential to overcome key limitations of surgical methods, which are associated with slow regeneration rates and insufficient efficacy in cases of extensive nerve defects [16]. The principal strategies of cell-based therapy for PNIs and other PNS disorders include the transplantation of SCs, application of MSCs, use of induced pluripotent stem cells (iPSCs), as well as combined approaches involving the use of cellular grafts together with nerve conduits and other biomaterials [71,147,148] (Figure 2).

### 4.1. Schwann Cells

Schwann cells (lemmocytes) are specialized glial cells of the PNS responsible for maintaining the structural integrity and functional activity of nerves, and they play a central role in nerve regeneration processes. Two major types of SCs are distinguished: myelinating SCs, which form the myelin sheath around axons, and non-myelinating SCs, which provide metabolic support and contribute to the maintenance of ionic homeostasis [149].

Following PNI, the distal nerve segment undergoes Wallerian degeneration, during which cellular debris is generated and subsequently phagocytosed by SCs and resident or recruited macrophages [150,151,152]. After debris clearance, the proximal axonal segment initiates regenerative outgrowth. At this stage, SCs undergo dedifferentiation and activation, begin to secrete neurotrophic factors, thereby creating a reparative microenvironment, and form so-called Bands of Büngner, which serve as guiding structures for regenerating axons [150,153,154].

In cases of extensive nerve damage, endogenous SC reactivation and the intrinsic regenerative capacity may be insufficient to prevent irreversible atrophy of denervated tissues before reinnervation is completed. Under such conditions, transplantation of exogenous SCs into the injury site is considered a promising therapeutic strategy to enhance the regenerative response [150,153].

One approach to cell therapy of the PNS involves the direct injection of an SC suspension into the site of injury. However, this method has several significant limitations: during injection, cells are exposed to substantial shear stress leading to cellular damage, and they exhibit low targeting efficiency and survival at the lesion site. Moreover, the unfavorable ischemic and inflammatory microenvironment of the injured tissue may cause the rapid loss of reparative properties and undesirable phenotypic alterations of transplanted cells [155,156].

Therefore, a more favorable strategy involves combined transplantation of SCs using nerve conduits or grafts, which provide a structural scaffold for cell adhesion, migration, and directed growth. The efficacy of SC transplantation has been extensively investigated in various animal models, where it demonstrated a pronounced stimulatory effect on peripheral nerve regeneration. In rodent studies, the use of cultured autologous SCs promoted enhanced axonal growth, remyelination, and successful repair of nerve defects, particularly when applied as part of tissue-engineered constructs. These encouraging preclinical results provided the rationale for the initiation of clinical trials. In 2012, the U.S. Food and Drug Administration (FDA) approved the first clinical application of autologous SCs for the treatment of spinal cord injury, paving the way for active investigation of their potential in human peripheral nerve regeneration [71,157,158].

#### Clinical Trials Using Schwann Cells

The first successful transplantation of autologous SCs in a human patient with severe PNI was performed in 2015 (Table 4). The reported clinical case described a female patient with complete transection of the sciatic nerve and a 7.5-cm nerve gap resulting from multiple deep lacerations of the posterior right thigh caused by contact with a motorboat propeller. Surgical treatment involved autologous sural nerve grafting combined with transplantation of autologous SCs seeded onto a biodegradable, biocompatible collagen matrix DuraGen. During the postoperative period, partial recovery of both sensory and motor functions was observed. Ultrasound examination and magnetic resonance imaging confirmed continuity of the reconstructed nerve trunk and the absence of tumor formation [159,160,161].

In 2017, the results of a second case involving the use of autologous SCs were reported in a female patient with a gunshot wound to the thigh and associated sciatic nerve injury. The therapeutic approach similarly included autologous sural nerve grafting in combination with SCs delivered on a collagen scaffold. Postoperatively, complete recovery of motor function in the tibial nerve-innervated region and partial restoration of sensory function were documented [155,159,161].

Based on the successful outcomes of these combined transplantation approaches, a Phase I clinical trial was initiated at the University of Miami to evaluate the safety of autologous SC transplantation in patients with severe PNI (nerve gaps of 5–10 cm) (NCT03999424). To date, the results of this study have not been published.

Despite these promising findings, the widespread clinical application of SC-based therapy for PNI faces several methodological limitations. These include the labor-intensive and time-consuming procedures required for cell harvesting and expansion, as well as the low survival rate and limited functional activity of a substantial proportion of transplanted cells. Prolonged in vitro cultivation is also associated with a reduced differentiation potential of SCs, an increased risk of glial scar formation, and insufficient secretion of neurotrophic factors. In turn, the use of allogeneic SCs carries the risk of developing immune responses, necessitating immunosuppressive therapy [71,153,155,170]. In light of these limitations, the therapeutic potential of alternative cell types for the treatment of PNI and other PNS disorders is being actively explored.

### 4.2. Mesenchymal Stromal Cells

Mesenchymal stromal cells represent a population of multipotent cells found in most postnatal tissues [171,172]. They were initially identified in the bone marrow, which remains the most extensively studied source. Subsequently, MSCs have been successfully isolated from a wide range of alternative tissues, including adipose tissue, umbilical cord blood, umbilical cord tissue (Wharton’s jelly), placenta, amniotic fluid, dental pulp, hair follicles, skin, and breast milk [148,173].

MSCs are characterized by high proliferative capacity, self-renewal ability, and multilineage differentiation potential, including differentiation into mesoderm-derived cell types (osteoblasts, chondrocytes, adipocytes, and myocytes), as well as into cells of other lineages, such as neurons, glial cells, and hepatocytes. These properties, together with pronounced neurotrophic activity, underlie the substantial potential of MSCs for applications in tissue engineering and regenerative medicine [148,174]. Of particular interest for clinical use are MSC sources that allow minimally invasive tissue harvesting. Currently, in addition to bone marrow, adipose tissue is considered one of the most favorable sources due to the high concentration of the stromal vascular fraction and the technical simplicity of aspiration procedures. Umbilical cord tissue and Wharton’s jelly also represent promising sources, as cell isolation from these tissues is ethically acceptable and does not involve invasive procedures for the donor [148,173].

The therapeutic potential of MSCs in PNI is associated with their paracrine and autocrine activity, as well as their high proliferative capacity. MSCs exhibit chemotactic migration toward the injury site, where they secrete a broad spectrum of biologically active molecules with antioxidant, anti-inflammatory, anti-apoptotic, antifibrotic, and antibacterial effects. This dynamic response to changes in the local microenvironment forms the basis of the reparative action of MSCs. Under the influence of local signals, MSCs are capable of promoting angiogenesis through direct cell–cell interactions and the secretion of VEGF, making them a valuable component for the vascularization of tissue-engineered constructs [175,176,177].

A key mechanism in peripheral nerve regeneration is the ability of MSCs to secrete neurotrophic factors such as NGF, GDNF, BDNF, CNTF, and neuregulin-1 [175]. These factors exert neuroprotective effects, prevent axonal degeneration, and stimulate growth cone activity. In addition, MSCs modulate the immune response by reducing the intensity of inflammation at the injury site. In experimental animal models of sciatic nerve injury, MSC transplantation promoted accelerated axonal regeneration and remyelination, as well as a reduction in neuropathic pain severity [176,178].

#### 4.2.1. Preclinical Studies of MSC-Based Therapy for Peripheral Nerve Injury

The efficacy of MSC-based therapy for PNI has been extensively investigated in preclinical models. Various strategies have been employed to deliver MSCs to the site of injury, including systemic (intravenous) and local (intramuscular, epineurial, or subepineurial) administration, as well as the implantation of cells within nerve or muscle grafts [148].

##### Bone Marrow-Derived Mesenchymal Stromal Cells

Bone marrow-derived mesenchymal stromal cells (BM-MSCs) are among the most extensively studied adult stem cell populations. As multipotent cells, BM-MSCs are capable of differentiating into various cell types, including neurons and SC-like cells. BM-MSCs are characterized by the relatively labor-intensive harvesting process, which yields a limited number of cells, and the painful nature of bone marrow aspiration. Moreover, the proliferative capacity of these cells is largely determined by the post-transplantation microenvironment. Nevertheless, multiple studies have demonstrated their stimulatory potential in peripheral nerve regeneration following injury. It has been shown that approximately 5% of transplanted BM-MSCs undergo transdifferentiation into SCs after transplantation into the injured nerve [179]. Numerous studies across different animal species and PNI models have confirmed the regenerative potential of BM-MSCs (Table 5).

The mechanisms underlying the regenerative effects of BM-MSCs are thought to involve both the direct expression of neurotrophic factors and indirect modulation of endogenous SCs. Depending on the number of transplanted cells, BM-MSCs enhance myelination and stimulate axonal growth from the proximal to the distal nerve segments, including participation in the formation of Bands of Büngner. When transplanted into the injured brain, BM-MSCs have been shown to express neuronal markers, such as neuronal nuclei antigen (NeuN), as well as the astrocytic marker glial fibrillary acidic protein (GFAP) [217]. Studies have also demonstrated improved peripheral nerve regeneration following BM-MSC transplantation both directly into the nerve and when incorporated into various nerve conduits (Table 5). In addition, BM-MSCs promoted angiogenesis and exerted neuroprotective effects in models of PNI in small animals [218].

For example, a comparative analysis of the efficacy of intravenous versus epineurial delivery of BM-MSCs was performed in rats with sciatic nerve injury and following hind limb transplantation. Administration of BM-MSCs enhanced nerve regeneration in both models. Notably, local cell delivery promoted structural recovery, as evidenced by an increased number of axons, whereas systemic administration resulted in greater improvements in electrophysiological and functional outcomes [181]. In another study, a combined strategy involving BM-MSC transplantation together with immunomodulatory therapy demonstrated superior functional recovery after complete sciatic nerve transection in rats, which was attributed to improved survival of transplanted cells under immunomodulatory conditions [182,219].

The efficacy of MSC-based therapy has been demonstrated not only in rodent models but also in large-animal models. In a rabbit model of acute and subacute sciatic nerve injury, both allogeneic BM-MSCs and their conditioned medium were evaluated. Local administration of either the cells or their secretome accelerated nerve regeneration, with a more pronounced effect observed during the subacute phase of injury [186]. Restoration of the radial nerve following a critical-sized defect (2.5 cm) was also assessed in non-human primates using several reconstruction approaches, including autologous nerve grafts, acellular allografts, and acellular allografts recellularized with autologous BM-MSCs. The best functional and morphological outcomes were achieved with acellular allografts recellularized with autologous BM-MSCs [216,220].

##### Adipose Tissue-Derived Mesenchymal Stromal Cells

Adipose tissue-derived mesenchymal stromal cells (AD-MSCs) exhibit multipotency, multilineage differentiation capacity, and substantial therapeutic potential [172]. Of particular interest in the context of functional recovery after PNI is their ability to undergo neurogenic differentiation. AD-MSCs are known to possess high proliferative capacity and a greater differentiation potential compared with BM-MSCs [221]. The use of SC precursors derived from human AD-MSCs within a polymeric conduit to bridge a 13-mm sciatic nerve defect in rats resulted in significant functional recovery comparable to that achieved with autologous nerve grafting [211]. Administration of AD-MSCs preconditioned with resveratrol to enhance their regenerative capacity in a compression-induced PNI model led to accelerated functional recovery, increased motor neuron counts, and reduced muscle atrophy [189].

AD-MSCs secrete a variety of growth factors, some of which are present at higher levels than those produced by other stem cell populations, including embryonic stem cells and amniotic cells. These findings suggest that AD-MSC-mediated peripheral nerve regeneration is, at least in part, driven by the paracrine secretion of trophic factors, independent of donor age or anatomical origin [222].

An additional reparative mechanism of AD-MSCs may be related to their ability to differentiate into endothelial cells, thereby indirectly promoting angiogenesis at the injury site [223]. Supporting this notion, the application of canine AD-MSCs in a rat model of sciatic nerve compression injury resulted in accelerated functional and electrophysiological recovery [188].

Particular interest has been directed toward the AD-MSC secretome, which exhibits a strong capacity to establish a regenerative microenvironment. Several studies have evaluated the regenerative potential of AD-MSCs and their conditioned medium in a rat model of inflammatory sciatic nerve injury. The results demonstrated that both AD-MSCs and their conditioned medium independently exert pronounced neuroregenerative and neuroprotective effects [192] (Table 5).

##### Dental Pulp-Derived Mesenchymal Stromal Cells

Dental pulp-derived mesenchymal stromal cells (DP-MSCs) represent a population of multipotent cells that express neural lineage markers and possess the capacity to secrete neurotrophic factors. DP-MSCs have been shown to differentiate in vitro into an SC-like phenotype and to support the survival and axonal outgrowth of dorsal root ganglion neurons [224].

Results from several studies using rat models of sciatic nerve injury have confirmed the neuroprotective effects of DP-MSCs, manifested by the enhanced survival of dorsal root ganglion neurons following injury [195,196,206]. In particular, when DP-MSCs were incorporated into fibrin glue within a collagen conduit, a significant increase in the number of axons in the distal nerve segment was observed after 12 weeks, along with an elevated expression of markers associated with myelination and angiogenesis, indicating active nerve regeneration [195]. In a model of a critical-sized 15-mm sciatic nerve defect repaired using a NeuraWrap™ collagen conduit, transplantation of DP-MSCs promoted axonal regeneration, remyelination, and neovascularization [214]. It has also been demonstrated that the transplantation of SC-like cells derived from DP-MSCs within a collagen conduit facilitated regeneration of the rat sciatic nerve following a 6-mm defect [198]. In a compression-induced PNI model, DP-MSCs administered either alone or in combination with BM-MSCs exhibited pronounced neuroregenerative potential, as evidenced by progressively improving sciatic functional index (SFI) values throughout the observation period [190]. In another study, exosomes isolated from human DP-MSCs were shown to stimulate SC proliferation and migration in vitro and enhance axonal regeneration, resulting in improved functional recovery in a rat model of sciatic nerve injury [191].

Collectively, these experimental data indicate that DP-MSCs represent an effective cellular resource capable of significantly enhancing reparative processes following PNI (Table 5).

##### Human Umbilical Cord- and Wharton’s Jelly-Derived Mesenchymal Stromal Cells

The use of mesenchymal stromal cells derived from the human umbilical cord (UC-MSCs) and Wharton’s jelly (WJ-MSCs) represents a promising approach in PNS regenerative therapy. UC-MSCs are capable of differentiating into SC-like cells and expressing neurotrophic factors such as NGF and BDNF [181]. Transplantation of these cells following sciatic nerve transection in rats promoted motor function recovery and the formation of Büngner bands [207].

Applying UC-MSCs within an amniotic membrane for the repair of 6–8 mm sciatic nerve defects resulted in significant improvements in functional, electrophysiological, and histological outcomes [225]. Injection of UC-MSCs into a nerve crush injury site stimulated axonal regeneration and increased the expression of *BDNF* and its receptor TrkB [226]. In a recent study, bio-3D conduits constructed from UC-MSCs were evaluated in a rat model of PNI. The results demonstrated that UC-MSC-derived bio-3D conduits supported robust axonal regeneration comparable to that achieved with autologous nerve grafts [197].

WJ-MSCs have likewise been shown to differentiate into SC-like phenotypes and to secrete neurotrophic factors. Their application promoted remyelination and functional recovery following sciatic nerve crush injury, although the regenerative effect was attenuated when combined with a chitosan membrane [227]. When incorporated into a polycaprolactone conduit to bridge a 10-mm sciatic nerve defect, WJ-MSCs significantly enhanced nerve regeneration through the increased expression of trophic factors, including BDNF, netrin-1, GDNF, VEGF, and angiopoietin-1 [228]. In a 10-mm sciatic nerve defect model, WJ-MSCs were shown to improve peripheral nerve regeneration predominantly via paracrine and immunomodulatory mechanisms [208] (Table 5).

#### 4.2.2. Clinical Studies of Mesenchymal Stem Cell-Based Therapies

In 2022, a Phase I clinical trial (NCT05333406) was completed to evaluate the safety, tolerability, and determine the maximum tolerated dose of EN001—an allogeneic UC-MSC-based therapy—in nine patients with CMT1A (Table 4). According to press releases, no serious adverse events have been reported [162]. Given the limited sample size of the Phase I study, further safety evaluation of EN001 is ongoing in a Phase Ib trial involving 12 patients with CMT1A (NCT06328712); results have not yet been published. Another study (NCT06218134) investigated the efficacy of EN001 in patients with CMT1E; however, the results have not yet been published.

In 2024, a Phase I clinical trial (NCT05947578) assessing the intramuscular administration of CLZ-2002 in patients with CMT1A was completed. CLZ-2002 consists of allogeneic tonsil-derived MSCs induced toward an SC-like phenotype [229,230]. To date, official results of this study have not been published.

The therapeutic potential of MSCs has also been explored in diabetic neuropathy. In a clinical trial completed in 2016 (NCT02387749), intravenous administration of autologous BM-MSCs in patients with diabetic neuropathy was not associated with serious adverse events and demonstrated the ability to promote the biological recovery of peripheral nerves [163,164,165].

In recent years, additional clinical trials have been initiated to assess the safety and efficacy of MSCs derived from various sources, including bone marrow, umbilical cord tissue, and adipose tissue, in PNIs and neurological disorders (NCT04654286, NCT03336996, NCT04346680, NCT02853942). However, at present, no official results from these studies have been reported [231].

### 4.3. Other Cell Types

#### 4.3.1. Induced Pluripotent Stem Cells

Induced pluripotent stem cells (iPSCs) are considered a promising tool in cell-based therapies due to their ability to differentiate into a wide range of somatic cell types. They exhibit gene expression signatures, epigenetic profiles, and differentiation potential comparable to those of embryonic stem cells (ESCs) while circumventing the ethical concerns associated with ESC use [232,233]. A major challenge in the therapeutic application of iPSC-derived products remains safety, as conventional reprogramming methods involve the use of oncogenes (e.g., c-Myc). Nevertheless, alternative protocols have been developed to generate iPSCs without c-Myc, employing alternative transcription factors and nonviral delivery methods [234,235]. iPSCs generated using these approaches retain self-renewal capacity, display high proliferative activity, and preserve multilineage differentiation potential toward derivatives of all three germ layers [236].

It has been demonstrated that neural crest stem cells derived from human iPSCs, when incorporated into tissue-engineered conduits, accelerate remyelination and regeneration of the rat sciatic nerve [237]. iPSC-derived MSCs, in combination with acellular nerve grafts, promoted axonal regeneration and functional recovery in a 10-mm nerve defect model [209]. iPSC-derived neurospheres seeded onto the surface of polycaprolactone conduits stimulated axonal growth and supported functional restoration [238]. Similarly, biodegradable conduits containing iPSC-derived neurospheres in combination with FGF2 also demonstrated enhanced axonal regeneration [239].

Despite these encouraging results, the persistence of epigenetic memory related to the tissue of origin and the risk of chromosomal aberrations remain significant concerns, as iPSCs may be more susceptible to uncontrolled mutations than ESCs [240]. To date, no clinical trials have been registered evaluating iPSC-derived products for the treatment of PNS injuries in humans, although their therapeutic potential is being actively investigated in other indications, including retinal disorders, Parkinson’s disease, and spinal cord injury [241].

#### 4.3.2. Fibroblasts

Fibroblasts, together with SCs, play a crucial role in peripheral nerve regeneration [242]. It has been shown that when chitosan-based nerve conduits are used, fibroblasts are the first cells to colonize the lumen of the conduit, where they express neuregulin-1 and establish a signaling microenvironment that promotes the recruitment and activation of SCs [243]. By participating in the synthesis of extracellular matrix components and the basal lamina, fibroblasts contribute to the maintenance of tissue structural integrity. However, during regeneration, they may also promote excessive scar formation, thereby impairing functional recovery. Following peripheral nerve injury, fibroblasts migrate into the lesion site, forming a connective tissue bridge between the nerve stumps and providing a scaffold for subsequent SC migration and axonal outgrowth [244]. Recent evidence suggests that fibroblasts can be reprogrammed into SCs or Schwann-like cells through genetic approaches and epigenetic modulation [244,245].

In a mouse model of sciatic nerve injury, SCs derived from human fibroblasts were employed. These transdifferentiated cells exhibited characteristic morphological, molecular, and functional features of SCs, and their application promoted nerve regeneration and functional recovery [245]. A comparative analysis of fibroblasts from different tissue sources demonstrated that fibroblasts derived from peripheral nerves possess a greater capacity to stimulate neurite outgrowth of dorsal root ganglion neurons than dermal fibroblasts. In a rat model of PNI, nerve-derived fibroblasts created a more favorable paracrine microenvironment for regeneration [246].

In a single-center, open-label, uncontrolled study conducted in Japan, the safety of Bio-3D nerve conduits fabricated from autologous fibroblasts was evaluated for peripheral nerve repair in the hand. During a 48-week follow-up of three patients, no adverse events or signs of graft rejection were reported. All participants demonstrated recovery of sensory and motor function within the innervated area [166] (Table 4).

#### 4.3.3. Bone Marrow Mononuclear Cells

Bone marrow mononuclear cells (BMMNCs) constitute a heterogeneous cell population comprising endothelial progenitor cells, MSCs, and HSCs. Their advantage in cell-based therapy lies in the relative ease of isolation from bone marrow using density gradient centrifugation [233]. BMMNCs exhibit pronounced immunomodulatory and neuroprotective properties mediated by the secretion of a broad spectrum of trophic factors and cytokines [171]. Their efficacy has been demonstrated in various experimental models of nerve injury, including transection, compression, and diabetic neuropathy [247].

In a mouse model of sciatic nerve compression injury, systemic administration of BMMNCs promoted axonal regeneration and remyelination and exerted immunomodulatory effects [247]. Studies investigating the therapeutic time window revealed that both early (within 7 days) and delayed (after 7 days) administration of BMMNCs following sciatic nerve compression in rats improved axonal regeneration and myelination, which correlated with the recovery of motor and sensory functions [248].

Clinical studies evaluating BMMNCs in peripheral nerve disorders remain limited. In a retrospective study involving 44 patients with median or ulnar nerve injuries, reconstruction using silicone conduits filled with autologous BMMNCs resulted in more effective nerve regeneration compared with empty conduits [167]. In patients with diabetic neuropathy, intramuscular administration of autologous BMMNCs in cases of refractory disease led to improvements in motor and sensory function, stimulation of angiogenesis, and enhanced neuroregeneration [168,169] (Table 4).

#### 4.3.4. Neural Stem Cells

Neural stem cells (NSCs) represent a self-renewing cell population capable of giving rise to all neuronal and glial cell types of both the CNS and PNS. NSCs were first isolated from the adult mouse brain in 1992 [249], and were subsequently identified in other species, including primates. In mammals, the main NSC niches are located in the hippocampus and adjacent regions of the striatum [250]. Despite methodological challenges associated with cell harvesting and in vitro expansion, NSCs demonstrate a high regenerative potential in models of PNI.

In a porcine femoral nerve reconstruction model using an autologous venous conduit coated with hippocampal NSCs, a significant enhancement of nerve regeneration was observed over a 6-month period compared with the controls. The therapeutic effect was shown to be partially mediated by the regulation of cyclic nucleotide phosphodiesterase expression, leading to activation of endogenous SCs [251].

The combination of murine NSCs with FGF1 within a poly-D,L-lactic acid conduit for bridging a 15-mm sciatic nerve defect in rats accelerated axonal outgrowth and promoted the early recovery of motor function [252]. Under similar conditions, immortalized human NSCs stimulated neovascularization, axonal regeneration, and muscle reinnervation [253]. Recent studies have increasingly focused on the regenerative potential of cell populations exhibiting properties closely related to NSCs. These include neuroepithelial stem cells (NESCs) and induced neural progenitor cells (iNPCs). In a rat model of a critical (10-mm) sciatic nerve defect, transplantation of NESCs directly into the injury site resulted in a statistically significant stimulation of axonal regeneration [254].

In another study, the use of iNPCs also demonstrated a positive therapeutic effect, manifested as significant functional recovery. However, the authors noted that the level of integration and long-term survival of transplanted cells within host tissues remained low. This finding suggests that paracrine mechanisms—such as the secretion of neurotrophic and anti-inflammatory factors—may play a more prominent role than direct cell replacement in mediating the therapeutic outcome [255]. These results highlight the promise of stem cell-derived populations for nerve repair, while simultaneously emphasizing the need to improve their survival and integration within recipient tissues.

A key limitation of NSC-based therapies remains the risk of oncogenesis, including the formation of neuroblastomas [256], which currently hinders their translation into clinical applications.

#### 4.3.5. Skin-Derived Stem Cells

Skin-derived stem cells (SDSCs), localized within the dermis, represent an accessible source of adult stem cells. These multipotent cells exhibit high proliferative activity in vitro and are capable of differentiating into a broad range of cell types, including melanocytes, chondrocytes, osteocytes, connective tissue cells, endocrine cells, as well as neurons and glial cells of the autonomic and PNS [257].

It has been demonstrated that culturing SDSCs in media supplemented with neuregulin-1 induces their differentiation into SC-like cells expressing key markers of peripheral nerve regeneration, such as S100, myelin basic protein (MBP), GFAP, and p75. Transplantation of SCs derived from SDSCs into a sciatic nerve crush injury site was shown to enhance axonal remyelination [258]. In a sciatic nerve transection model, SDSC-derived SCs promoted the recovery of thermal sensitivity and increased the survival of sensory neurons [259]. Intraneural administration of SDSCs in combination with antibodies against interleukin-6 (IL-6) did not result in a significant increase in macrophage infiltration or myelin debris clearance. However, this treatment led to the reduced expression of inducible nitric oxide synthase (iNOS), indirectly indicating an improvement in axonal regeneration. [260,261]. The use of SCs differentiated from skin progenitor cells (SPC-SCs), together with their extracellular matrix incorporated into a tissue-engineered nerve conduit, resulted in enhanced peripheral nerve regeneration and functional recovery [262]. Earlier studies also demonstrated that exosomes derived from SPC-SCs possess neuroregenerative potential in animal models of PNI, stimulating axonal growth and myelination [263].

#### 4.3.6. Hair Follicle Stem Cells

Hair follicle stem cells (HFSCs) are pluripotent cells isolated from the hair bulb region of adult mammals. These cells exhibit high proliferative activity in vitro and express ESC-associated markers, including Nanog, Oct4, and nestin. HFSCs demonstrate the capacity to differentiate into multiple cell lineages, such as adipocytes, myocytes, melanocytes, epithelial and endothelial cells, as well as neurons and glial cells. However, their proliferative capacity is limited, which restricts long-term in vitro expansion [264].

Despite this limitation, several studies have reported successful applications of HFSCs in peripheral nerve regeneration. Human HFSCs transplanted into nerve injury sites were shown to differentiate into GFAP-positive cells and promote the functional recovery of denervated limbs [265]. In another study, the combined use of SCs and neurons derived from HFSCs within a decellularized nerve conduit demonstrated cell survival for up to 8 weeks and improved electrophysiological parameters of nerve regeneration [266]. The application of HFSCs encapsulated within a polyvinyl membrane in a murine sciatic nerve injury model further confirmed their ability to differentiate into neuronal and glial cells [267]. In a separate study, HFSCs isolated from rat whisker follicles stimulated axonal growth and improved functional recovery following PNI, as assessed by histological and electrophysiological analyses [268]. More recent studies have shown that small extracellular vesicles derived from hair follicle neural crest stem cells enhance axonal regeneration and reduce muscle atrophy in a sciatic nerve defect model [269].

#### 4.3.7. Skeletal Muscle-Derived Stem Cells

Skeletal muscle-derived stem cells (SMDSCs) constitute a multipotent cell population with high proliferative capacity and self-renewal ability. In culture, these cells exhibit a rounded morphology and form spherical colonies. A notable advantage of SMDSCs is their resistance to oxidative and hypoxic stress. Their ability to differentiate into neuronal cells has been demonstrated, along with their pronounced secretory activity. Following muscle injury, SMDSCs release trophic factors involved in reinnervation processes [270].

Undifferentiated murine SMDSCs transplanted into a sciatic nerve crush injury site were shown to differentiate in vivo into SCs, perineurial, endoneurial, and endothelial cells, thereby promoting nerve regeneration, remyelination, and vascularization [271]. In a subsequent study, the same authors employed human SMDSCs to repair a 12-mm crush injury and a 7-mm sciatic nerve defect using a decellularized nerve conduit, confirming the high regenerative potential and multilineage differentiation capacity of these cells [272]. Comparable results were reported by other researchers in rat sciatic nerve transection models followed by end-to-end suturing [273,274]. In another study, therapy with induced SMDSCs demonstrated preservation of the SC phenotype, reduction in muscle atrophy, and enhanced nerve regeneration [275]. In a large-animal model of PNI using mini pigs, implantation of SMDSCs into a collagen conduit resulted in functional recovery and neovascularization. Importantly, these cells exhibited low immunogenicity, suggesting their suitability for family-based allogeneic cell therapy [276].

### 4.4. Achievements and Limitations of Cell Therapy for Peripheral Nervous System Injuries and Disorders

Thus, preclinical and clinical studies indicate that cell-based therapies can enhance axonal regeneration, remyelination, angiogenesis, and functional recovery in PNS injuries through trophic support, immunomodulation, and structural guidance. Importantly, optimal therapeutic outcomes often require combining transplanted cells with nerve conduits or other biomaterial scaffolds, which provide mechanical support, enhance cell survival, and create a microenvironment for directed axonal growth. The use of MSCs, both autologous and allogeneic, has demonstrated therapeutic efficacy, partly due to their relatively low immunogenicity. In contrast, the transplantation of other allogeneic cell types generally requires concomitant immunosuppressive therapy to prevent immune rejection and ensure graft survival [165,170]. However, widespread clinical translation remains limited by challenges including low cell survival and engraftment efficiency, risk of immune rejection or tumorigenicity (especially for iPSC-derived products), labor-intensive manufacturing and expansion procedures, variability of therapeutic potency, and the need for robust long-term safety and efficacy data.

## 5. Gene-Cell Therapy

Genetically modified cells represent a promising approach for peripheral nerve regeneration. Among the most widely studied cell types are SCs and MSCs, which have been genetically engineered to enhance the secretion of neurotrophic factors and establish a pro-regenerative microenvironment at the injury site [71,277,278].

SCs were transduced with a lentiviral vector carrying the *GDNF* gene under the control of a doxycycline-inducible promoter. Transplantation of these genetically modified SCs into a rat model of PNI enabled localized, inducible GDNF expression, which stimulated axonal outgrowth and accelerated nerve regeneration [279].

Efforts have also been made to generate SCs capable of secreting multiple neurotrophic factors simultaneously. Since conventional genetic modification strategies typically allow the overexpression of only a single neurotrophin, a transcription factor-based approach was employed using the c-Jun gene. Lentiviral transduction of SCs resulted in the increased synthesis and secretion of GDNF, BDNF, and NGF. Co-culture of genetically modified and native SCs revealed enhanced migration and proliferation of the latter [280].

In another study, SCs were transduced with a lentiviral vector encoding *FGF2*. Following confirmation of *FGF2* overexpression in vitro, the modified SCs were embedded in silicone tubes and implanted into the injured sciatic nerve of rats. This gene-cell therapy approach led to significant functional recovery and an increased number of distal sensory neurons [121]. Similarly, SCs genetically engineered to express *VEGF* were incorporated into a nerve conduit. Evaluation of this construct in a critical (10-mm) sciatic nerve defect model demonstrated that VEGF-expressing SCs not only provided structural support for regenerating axons but also generated a robust pro-angiogenic and neurotrophic microenvironment via localized VEGF secretion. As a result, regenerative outcomes were comparable to those achieved with the current “gold standard”—autologous nerve grafting [281].

MSCs possess anti-inflammatory and neuroprotective properties and are therefore considered promising candidates for the treatment of neuropathic pain. MSCs were transduced with a retroviral vector carrying the human preproenkephalin (hPPE) gene. Intrathecal administration of these genetically modified MSCs to rats with a chronic sciatic nerve compression model resulted in reduced pain sensitivity concomitant with sustained *hPPE* expression [282]. In another study, MSCs were modified using an adenovirus carrying the gene for sirtuin 1 (SIRT1), which encodes an nicotinamide adenine dinucleotide (NAD^+^)-dependent histone deacetylase that modulates inflammation, oxidative stress, and mitochondrial function, thereby contributing to pain alleviation [283]. Intrathecal transplantation of these cells provided an analgesic effect by suppressing inflammation and enhancing neuroprotection [284].

The use of baculoviral vectors incorporating a Cre/loxP system to integrate the *GDNF* gene into MSCs enabled stable protein expression, which promoted SC recruitment to the injury site, enhanced axonal regeneration, and improved motor function in a rat model of PNI [285]. In another study, genetic modification of MSCs to overexpress the *TrkA* increased cell survival, promoted differentiation into SC-like cells, and enhanced axonal growth and remyelination following transplantation into nerve allografts [286]. Overexpression of *VEGF* in MSCs was also shown to be effective, resulting in nerve regeneration, increased neurotrophin secretion, and improved motor function in mice with PNI [287].

The regenerative potential of other genetically modified cell types has also been demonstrated. Human ESCs with inducible *FGF2* expression supported nerve regeneration and stimulated sensory fiber growth in murine models of PNI [288]. Furthermore, genetically modified NSCs expressing either *GDNF* or *BDNF* significantly enhanced axonal regeneration and myelination following critical sciatic nerve injury in rats, leading to improved functional recovery [289].

Human muscle progenitor cells were genetically modified to overexpress a combination of neurotrophic factors: BDNF, GDNF, VEGF, and IGF1. Injection of these modified cells into mice with PNI resulted in a significant reduction in both motor and sensory deficits, thus, demonstrating the synergistic efficacy of the long-term, combined delivery of neurotrophic molecules via cellular carriers [290].

A more advanced tissue-engineering strategy was implemented through the development of three-dimensional peripheral nerve tissueoids. These constructs combined *NT-3*-expressing SCs with a decellularized optic nerve scaffold serving as a guiding matrix for regeneration. The tissueoids were shown to effectively recruit TrkC-positive SCs, activate the PI3K/Akt signaling pathway in target neurons, and consequently promote accelerated reinnervation accompanied by the recovery of both sensory and motor functions in animal models [291]. Collectively, these studies underscore the substantial potential of combinatorial strategies integrating the genetic modulation of cells with advanced scaffold technologies to achieve functionally meaningful peripheral nerve regeneration.

Genetically modified cell-based therapies demonstrate strong regenerative potential in PNS injury models by enabling sustained, localized delivery of neurotrophic and immunomodulatory factors, resulting in enhanced axonal growth, remyelination, angiogenesis, pain reduction, and functional recovery. Nevertheless, their clinical translation is constrained by significant challenges, including biosafety concerns related to genetic modification, control and durability of transgene expression, manufacturing complexity, and the lack of long-term in vivo safety and efficacy data.

## 6. Conclusions

Injuries and diseases of the PNS remain a major medical challenge, frequently resulting in persistent functional deficits and a substantial reduction in patients’ quality of life. Conventional therapeutic approaches, including microsurgical reconstruction, physiotherapy, and symptomatic pharmacological treatment, have significant limitations: they often fail to achieve complete functional recovery, particularly in cases of extensive nerve defects or progressive neurodegenerative processes, and may be associated with adverse side effects. In this context, regenerative medicine strategies aimed at addressing the fundamental pathogenic mechanisms of PNS damage are becoming critically important for the future of PNS therapy.

Advances in molecular and cellular biology have given rise to two complementary therapeutic directions: gene therapy and cell therapy. Gene therapy, based on the use of viral (AAV, lentiviral) and non-viral (plasmid) vectors, enables the targeted delivery and sustained local expression of therapeutic genes encoding neurotrophic factors such as NGF, BDNF, GDNF, VEGF, and others. This approach overcomes key limitations associated with recombinant protein administration, including short half-life and systemic side effects. Successful outcomes in preclinical models—demonstrating enhanced axonal regeneration, remyelination, and functional recovery—are increasingly reflected in a growing number of clinical studies, including those targeting diabetic neuropathy and inherited disorders such as CMT disease.

Future directions should include the identification of novel disease-specific molecular targets and a deeper elucidation of the mechanisms underlying PNS injury, including axonal degeneration, neuroinflammation, and maladaptive neuronal hyperexcitability. In inherited neuropathies, emerging strategies such as gene replacement, gene silencing (e.g., RNA interference), and precise gene correction (e.g., CRISPR/Cas-based approaches) hold particular promise. Additionally, targeting molecular pathways associated with neuropathic pain—including ion channels and mediators of neuronal hyperexcitability—may reduce chronic pain following PNI.

In parallel, cell-based therapies have rapidly evolved, encompassing a wide range of cell types, from autologous SCs and MSCs derived from various tissues to iPSC-derived populations. Transplanted cells may not only replace lost components of neural tissue but, importantly, establish a robust paracrine regenerative microenvironment through the secretion of neurotrophic, angiogenic, and immunomodulatory factors. The integration of cellular grafts with tissue-engineered constructs—such as biodegradable nerve conduits and hydrogels—substantially enhances cell survival and spatially directed activity, bringing regenerative outcomes closer to the current “gold standard” of autologous nerve grafting.

The most promising and logically consistent evolution of these strategies is their convergence within gene-cell therapy. Genetic modification of cellular carriers, including SCs, MSCs, and muscle progenitor cells, to enable controlled overexpression of one or multiple neurotrophic factors effectively creates localized “biofactories” at the site of injury. Such approaches provide prolonged, dose-controlled, and combinatorial therapeutic effects, thereby overcoming the limitations inherent to both cell transplantation and single-dose gene delivery.

Despite the impressive progress achieved to date, several critical challenges must be addressed before these technologies can be broadly implemented in clinical practice. These include the optimization of vector safety and targeting specificity in gene therapy, enhancement of the survival and functional integration of transplanted cells, standardization of protocols for cell isolation, expansion, and genetic modification, and the execution of large-scale, well-controlled clinical trials to establish long-term efficacy and safety. Particular emphasis should be placed on personalized therapeutic strategies that account for the etiology and severity of PNS injury.

In summary, contemporary gene and cell therapy strategies open fundamentally new avenues for the pathogenetic treatment of PNIs and disorders, shifting the therapeutic paradigm from palliative symptom management toward active regeneration and functional restoration. Continued interdisciplinary research at the intersection of gene engineering, cell biology, and tissue engineering holds strong promise for the development of effective clinical solutions capable of markedly improving the outcomes for patients with peripheral nerve pathologies.

## Figures and Tables

**Table 1:** Current therapeutic approaches for peripheral nerve injuries, neuropathies, and peripheral nervous system disorders and their limitations.

Therapeutic Approach		Advantages	Limitations	References
Surgical Approaches	Neurorrhaphy (direct suturing of the proximal and distal nerve ends)	Applicable for short nerve gaps (<1 cm); promotes nerve regeneration	Does not guarantee complete functional recovery; regeneration is limited by a narrow therapeutic time window	[8,16,17]
	Autologous nerve grafting (transplantation of the patient’s own nerve)	Applicable for nerve defects >3 cm; autografts provide a natural scaffold for axonal growth due to preserved endoneurial architecture, SCs, adhesion molecules, and neurotrophic factors	Limited availability of donor tissue; the need for a second surgical procedure; the risk of pain, sensory loss, and neuroma formation at the donor site	[8,16,18,19,20]
	Allogeneic nerve grafting (transplantation of a donor nerve)	Applicable for nerve defects >3 cm; preserved donor endoneurium facilitates axonal regeneration, while the donor’s SCs promote remyelination	Requires prolonged immunosuppressive therapy (approximately 24 months) to prevent graft rejection	[8,16,21,22]
	Nerve transfer (neurotization)	Applicable for severe PNIs; rapid functional recovery; the procedure is performed outside the zone of scarring and injury; preservation of native nerve anatomy and biomechanics	The technique demands exceptional surgical expertise; high cost; limited donor nerve pool; the risk of donor site morbidity	[8,16]
	Nerve guidance conduits (tubular structures serving as a “bridge” between the proximal and distal nerve ends)	Elimination of donor-site morbidity; creation of an optimal microenvironment and a barrier against infiltration by surrounding tissues, as well as the ability to provide favorable conditions for cell adhesion, migration, and axonal growth	Limited efficacy in extensive nerve injuries and large nerve gaps; increased risk of misdirected or aberrant reinnervation during regeneration within hollow nerve conduits	[8,16,23,24,25]
Physiotherapy	Electrical stimulation	Effective in the correction of muscle atrophy; promotes nerve regeneration	Increases operative time and treatment costs and may reduce muscle excitability, decrease muscle fiber cross-sectional area, and impair neuromuscular junction integrity	[8,16,26,27]
	Ultrasound therapy	Promotes peripheral nerve regeneration by increasing axon number and diameter, enhancing myelination, accelerating nerve conduction velocity, and modulating the expression of neurotrophic factors	Clinical efficacy and optimal therapeutic protocols require further investigation	[26,28]
	Photobiomodulation	Promotes peripheral nerve repair through mitochondrial activation, stimulation of growth factor production (e.g., NGF), modulation of immune responses, and enhancement of angiogenesis	Primarily used as a rehabilitative modality; study outcomes remain inconsistent due to variability in irradiation parameters, and optimal treatment protocols have not yet been established	[26,29,30]
	Aerobic exercises	Increase blood flow, improve oxygen delivery, stimulate the release of neurotrophic factors, and activate neuronal pathways	Primarily used as a rehabilitative approach; limited evidence from well-controlled clinical studies	[26,31,32,33]
Pharmacotherapy	Erythropoietin	Exerts neuroprotective effects in PNIs by preventing axonal degeneration and neuronal apoptosis	Pronounced hematopoietic activity associated with an increased risk of thrombosis	[34]
	Steroid hormones	Improve sensory and motor recovery following PNIs; promote peripheral nerve regeneration	Serious adverse effects associated with long-term administration and high-dose use	[8,29,35]
	4-Aminopyridine	Enhances neuromuscular transmission; promotes remyelination; improves nerve conduction velocity	Clinical application is limited by a narrow therapeutic window and the risk of adverse effects	[8,29,36]

**Table 2:** Growth factors in peripheral nerve regeneration.

Growth Factor	Biological Functions	Localization of Expression (Primary Target Cells)	Advantages	Disadvantages	References
NGF	Stimulation of survival and neuritogenesis of sensory and sympathetic neurons; activation of SCs migration; modulation of nociception	Sensory neurons and their axons	High efficacy in sensory function recovery; promotes remyelination and contributes to restoration of motor function	Exacerbation of neuropathic pain under uncontrolled delivery; limited effects on motor neurons	[45,46,47,48,49]
BDNF	Support of motor neuron survival; regulation of myelination; stimulation of synaptic neurotransmitter synthesis; involvement in memory formation and synaptic plasticity	Widely expressed throughout the brain; motor neurons; SCs; a subset of dorsal root ganglion neurons	Key role in motor neuron maintenance and regeneration; enhancement of remyelination via SC activation	Rapid proteolytic degradation and short in vivo half-life; challenges in targeted delivery to motor neurons; elevated BDNF levels associated with chronic pain	[48,50,51,52,53,54]
GDNF	Potent survival factor for motor neurons and dopaminergic neurons; enhancement of post-injury myelination; support of SCs function	Motor neurons; astrocytes; oligodendrocytes; SCs	More pronounced effects on motor neurons compared with other growth factors; ability to prevent muscle atrophy following denervation	Risk of hypersensitivity during reinnervation	[48,55,56]
CNTF	Support of motor neuron survival; activation and proliferation of SCs	Myelinating SCs; glial cells	Effective in preventing motor neuron degeneration following axotomy	Pronounced systemic adverse effects (fever, anorexia, pain, muscle cramps) upon non-local delivery; low stability	[49,57,58,59]
FGF1, FGF2	Stimulation of angiogenesis; SCs proliferation and enhanced remyelination; neuronal repair	Broad range of cell types and tissues	Establishment of a favorable microenvironment through improved vascularization; accelerated SCs migration to the injury site; synergistic interactions with other growth factors	Pleiotropic effects affecting multiple cell types (risk of fibrosis); short half-life	[47,60,61,62,63]
IGF1	Stimulation of axonal growth; support of motor neuron survival; enhancement of remyelination	Inflammatory cells (e.g., macrophages); motor neurons in PNIs	Synergistic effects with other growth factors; acceleration of axonal regeneration and restoration of neuromuscular synapses	Potential stimulation of proliferation in non-target cell populations	[48,64,65,66,67,68]
VEGF	Potent stimulator of angiogenesis; direct neurotrophic and neuroprotective effects	Endothelial cells	Improved blood supply and oxygenation within the regenerative niche; direct stimulation of axonal growth and neuronal survival	Potential induction of undesirable angiogenesis	[48,60,69,70]
NT-3	Regulation of neuronal morphology; support of SCs survival and functional activity	Sensory and motor neurons; SCs	Maintenance of the reparative SCs phenotype	Short half-life	[48,71,72,73]
HGF	Induces neurite outgrowth in sensory neurons; promotes SCs migration and proliferation	Mesenchymal cells; endothelial cells	Modulation of angiogenesis; promotion of tissue protection and regeneration; induction of axonal growth	Inducer of lymphangiogenesis, angiogenesis, and tumor growth	[74,75,76,77]
TGF-β	Involved in the regulation of cell proliferation, differentiation, wound healing, and immune responses	Macrophage, SCs, fibroblasts	Establishment of a regenerative microenvironment; support of SCs survival; regulation of blood–nerve barrier permeability	Hyperactivation of signaling pathways leads to tumorigenesis, inflammation, fibrosis, and immunodeficiency	[78,79,80,81]
BMP	Involved in neurogenesis and neural repair; promotes SCs survival and proliferation	SCs	Activation of the reparative SCs phenotype; enhancement of axonal regeneration	Some family members inhibit axonal growth	[81,82,83,84,85]

**Table 3:** Clinical studies of gene therapy for peripheral nervous system disorders.

Condition	Drug	Phase/Status	Clinical Trial ID	Route of Administration	Outcome	References
Diabetic peripheral neuropathy	VM202 (Engensis); pDNA, encoding two isoforms of HGF	Phase II/ Completed	NCT01475786	Intramuscular	Demonstrated safety and efficacy, with significant pain reduction (up to 48% responders) and enhanced sensory function	[129]
		Phase III/ Completed	NCT02427464	Intramuscular	Pain reduction was observed; however, primary efficacy endpoints were not met. In an additional 12-month cohort, clinically meaningful pain reduction was noted. Further Phase III trials are warranted	[130]
Charcot–Marie–Tooth disease type 1A	VM202 (Engensis)	Phase I/II/ Completed	NCT05361031	Intramuscular	Demonstrated safety and good tolerability, with functional improvement in patients	[133]
	scAAV1.tMCK.NTF3	Phase I/II/ Not yet recruiting	NCT03520751	Intramuscular	Results not yet published	
Opioid-resistant pain in cancer patients	NP2; HSV-1, encoding the preproenkephalin gene	Phase I/ Completed	NCT00804076	Intradermal	Demonstrated safety and good tolerability, with dose-dependent reduction in numeric rating scale pain scores (up to ~80% decrease from baseline within 7–14 days)	[135,136,137]
		Phase II/ Completed	NCT01291901	Intradermal	Results not yet published	[135,136]
Adrenomyeloneuropathy	SBT101; AAV9, encoding the human *ABCD1* gene	Phase I/II/ Terminated	NCT05394064	Intrathecal	Demonstrated good tolerability; however, disease progression was observed in three patients following administration, warranting further monitoring	[139]
Giant axonal neuropathy	scAAV9/JeT-GAN	Phase I/Active, not recruiting	NCT02362438	Intrathecal	Improvement in motor function was observed with electrophysiologic stabilization and 3–48x increase in regenerating nerve clusters; however, adverse events and elevated anti-AAV9 antibody levels were reported	[141]
Krabbe disease	Combined approach: HSC transplantation followed by administration of AAVrh.10-hGALC (FBX-101)	Phase I/II Active, not recruiting	NCT04693598; children up to 12 months	Intravenous	Demonstrated safety and good tolerability	[145]
		Phase I/II/ Active, not recruiting	NCT05739643; children up to 18 years	Intravenous	Results not yet published	

**Table 4:** Clinical trials of cell therapy for peripheral nerve injuries and disorders.

Cell Type	Condition	Phase/Status	Clinical Trial ID	Route of Administration	Outcome	References
Autologous SCs	Complete transection of the sciatic nerve	–	–	Autologous sural nerve grafting combined with SCs seeded onto a collagen matrix DuraGen	Partial recovery of sensory and motor function	[159,160,161]
	Sciatic nerve injury	–	–	Autologous sural nerve grafting combined with SCs seeded onto a collagen matrix DuraGen	Complete recovery of tibial nerve motor function and partial restoration of sensory function	[155,159,161]
Autologous SCs	PNI	Phase I/ Completed	NCT03999424	Autologous sural nerve grafting combined with SCs seeded onto a collagen matrix DuraGen	Results not yet published	
Allogeneic UC-MSCs (EN001)	CMT1A	Phase I/ Completed	NCT05333406	Intravenous administration	Demonstrated safety	[162]
		Phase Ib/ Completed	NCT06328712	Intravenous administration	Results not yet published	
	CMT1E	Not applicable/ Completed	NCT06218134	Intravenous administration	Results not yet published	
Allogeneic MSCs derived from tonsillar tissue and induced to differentiate into Schwann cell-like cells (CLZ-2002)	CMT1A	Phase I/ Completed	NCT05947578	Intramuscular administration	Results not yet published	
Autologous BM-MSCs	Diabetic peripheral neuropathy	Not applicable/ Completed	NCT02387749	Intravenous administration	No serious adverse events were observed; BM-MSCs were shown to promote peripheral nerve regeneration	[163,164,165]
3D nerve conduit created from autologous patient-derived fibroblasts	PNI	–	–	Implantation at the injury site	Restoration of sensory and motor function without signs of graft rejection or adverse events	[166]
Silicone conduit filled with autologous BMMNCs	PNI	–	–	Implantation at the injury site	Enhanced nerve regeneration compared with empty silicone conduits	[167]
Autologous BMMNCs	Refractory diabetic sensorimotor polyneuropathy	–	–	Intramuscular administration	Symptom improvement with enhanced angiogenesis and neuroregeneration	[168]
		–	–	Intramuscular administration	Improvement in motor and sensory function	[169]

**Table 5:** Regenerative potential of mesenchymal stromal cells and their derivatives for the treatment of peripheral nerve injuries: preclinical studies in animal models.

Experimental Model	Cell Type/Delivery Method	Outcome	References
Transection	BM-MSCs + PLGA conduit	Improved SFI and histological outcomes	[180]
	BM-MSCs (injectable delivery)	Increased axonal number and improved electrophysiological and functional outcomes	[181]
	BM-MSCs + immunomodulation	Enhanced SFI, accelerated recovery of nerve T2 values, and restoration of nerve architecture	[182]
	AD-MSCs + fibrin glue	Enhanced myelination and stimulation of axonal regeneration and angiogenesis	[183]
Compression	BM-MSCs (injectable delivery)	Enhanced myelination, enhanced axonal outgrowth, and reactivation of endogenous SCs	[184]
	BM-MSCs in conditioned medium/injectable delivery	Enhanced myelination, improved axonal regeneration, and functional recovery	[185]
	BM-MSCs/conditioned medium	Functional, histological, and morphological recovery	[186]
	AD-MSCs/conditioned medium	Preservation of muscle tissue, increased SFI, and histological and functional recovery	[187]
	AD-MSCs (injectable delivery)	Increased SFI and accelerated functional recovery	[188]
	AD-MSCs preconditioned with resveratrol (injectable delivery)	Increased SFI, higher myelin sheath density, functional recovery, and increased motor neuron counts	[189]
	DP-MSCs/combined with BM-MSCs (injectable delivery)	Increased SFI, improved morphological parameters, and enhanced regenerative response	[190]
	DP-MSC-derived exosomes (injectable delivery)	Functional recovery with enhanced axonal regeneration and myelination	[191]
Inflammatory injury	AD-MSCs/conditioned medium	Increased SFI, improved nerve morphology, and enhanced axonal remyelination	[192]
5-mm nerve defect	SC–like cells derived from BM-MSCs/autologous decellularized graft	Histological and functional recovery	[193]
	AD-MSCs/gelatin hydrogel conduit	Enhanced myelination and axonal regeneration; improved motor function recovery; no in vivo differentiation into SCs observed	[194]
	DP-MSCs + fibrin glue/collagen conduit	Enhanced nerve regeneration and functional recovery	[195]
	DP-MSCs + collagen conduit	Increased angiogenesis, stimulation of SCs proliferation, and enhanced myelination	[196]
	Bio-3D conduit created from UC-MSCs	Functional recovery with enhanced myelination and axonal regeneration	[197]
6-mm nerve defect	SC-like cells derived from DP-MSCs + collagen conduit	Stimulation of axonal regeneration, increased myelination, and expression of canonical SC markers	[198]
8-mm nerve defect	BM-MSCs/autologous venous graft filled with Matrigel	No statistically significant improvement observed	[199]
	AD-MSCs/autologous vein filled with Matrigel		
10-mm nerve defect	BM-MSCs/chitosan conduit coated with poly(3-hydroxybutyrate)	Enhanced axonal regeneration	[200]
	BM-MSCs/decellularized nerve graft	Improved axonal regeneration and functional recovery	[201]
	AD-MSCs + fibrin glue/autologous nerve graft	Enhanced neuronal survival, vascularization and remyelination; recovery of myelin-associated gene expression	[202]
	Undifferentiated AD-MSCs and SC-like cells derived from AD-MSCs/biodegradable chitin conduit	Increased myelination, enhanced axonal regeneration, and neurotrophin secretion	[203]
	Neural cells derived from AD-MSCs/nanofibrous conduit	Histological and motor function recovery	[204]
	SC–like cells derived from AD-MSCs/fibrin conduit	Enhanced myelination	[205]
	DP-MSCs + fibrin conduit	Stimulation of axonal regeneration and neuroprotective effects on dorsal root ganglion neurons	[206]
	UC-MSCs/silicone conduit	Enhanced neovascularization and peripheral nerve regeneration	[207]
	WJ-MSCs + acellular nerve graft	Promotion of nerve regeneration with increased expression of neurotrophic and angiogenic growth factors	[208]
	iPSC-derived MSCs + acellular allogeneic nerve graft	Accelerated axonal regeneration, functional recovery, and stimulation of neovascularization	[209]
12-mm nerve defect	SC-like cells derived from BM-MSCs/chitosan conduit supplemented with Matrigel	Increased myelination and improved axonal regeneration	[210]
13-mm nerve defect	AD-MSCs/polymeric silicone conduit	Increased SFI, enhanced myelination, and improved sensory and motor function	[211]
15-mm nerve defect	AD-MSCs/polycaprolactone conduit containing hydrogel	Enhanced axonal regeneration and improved skeletal muscle regeneration	[212]
	AD-MSCs embedded in fibrin glue/laminin-based conduit	Promotion of neuronal regeneration	[213]
	DP-MSCs + NeuraWrap™ collagen conduit	Stimulation of angiogenesis and increased myelination	[214]
20-mm nerve defect	BM-MSCs/implant incorporating silicone tubes	Enhanced angiogenesis, preservation of muscle mass, and increased axonal regeneration	[215]
25-mm nerve defect	BM-MSCs + allogeneic nerve graft	Enhanced myelination, functional improvement, and increased nerve fiber diameter	[216]

## Data Availability

No new data were created or analyzed in this study. Data sharing is not applicable to this article.

## Abbreviations

The following abbreviations are used in this manuscript:

AAV	Adeno-associated virus
ABCD1	ATP-binding cassette subfamily D member 1
AD-MSC	Adipose tissue–derived mesenchymal stromal cell
Akt	Protein kinase B
ALS	Amyotrophic lateral sclerosis
ATP	Adenosine triphosphate
BDNF	Brain-derived neurotrophic factor
BM-MSC	Bone marrow-derived mesenchymal stromal cell
BMMNC	Bone marrow mononuclear cell
BMP	Bone morphogenetic protein
cDNA	Complementary DNA
CMT	Charcot–Marie–Tooth disease
CNS	Central nervous system
CNTF	Ciliary neurotrophic factor
DP-MSC	Dental pulp derived mesenchymal stromal cell
ERK	Extracellular signal-regulated kinase
ES	Electrical stimulation
ESC	Embryonic stem cell
FAK	Focal adhesion kinase
FDA	U.S. Food and Drug Administration
FGF	Fibroblast growth factor
GALC	Galactosylceramidase
GAN	Gigaxonin
GDNF	Glial cell line-derived neurotrophic factor
GFAP	Glial fibrillary acidic protein
HFSC	Hair follicle stem cell
HGF	Hepatocyte growth factor
HIV-1	Human immunodeficiency virus 1
hPPE	Preproenkephalin
HSC	Hematopoietic stem cell
HSV	Herpes simplex virus
IGF1	Insulin-like growth factor 1
IL	Interleukin
iNOS	Inducible nitric oxide synthase
iNPC	Induced neural progenitor cell
iPSC	Induced pluripotent stem cell
JAK	Janus-associated kinase
MAPK	Mitogen-activated protein kinase
MBP	Myelin basic protein
MSC	Mesenchymal stromal/stem cell
NAD	Nicotinamide adenine dinucleotide
NCAM	Neural cell adhesion molecule
NESC	Neuroepithelial stem cell
NeuN	Neuronal nuclei antigen
NGF	Nerve growth factor
NSC	Neural stem cell
NT-3	Neurotrofine-3
p75NTR	p75 neurotrophin receptor
pDNA	Plasmid DNA
PI3K	Phosphatidylinositol-3-kinase
PLGA	Poly(lactide-co-glycolide)
PMP22	Peripheral myelin protein 22
PNI	Peripheral nerve injury
PNS	Peripheral nervous system
SC	Schwann cell
SDSC	Skin-derived stem cell
SFI	Sciatic functional index
SIRT1	Sirtuin 1
SMAD	Suppressor of mother against decapentaplegic
SMDSC	Skeletal muscle-derived stem cell
SPS-SC	Schwann cell differentiated from skin progenitor cell
STAT	Signal transducer and activator of transcription
TGF	Transforming growth factor
Trk	Tyrosine kinase receptor
UC-MSC	Umbilical cord mesenchymal stromal cell
VEGF	Vascular endothelial growth factor
VSV-G	Vesicular stomatitis Indiana virus glycoprotein G
WJ-MSC	Warton’s jelly mesenchymal stromal cell
